# The complete chloroplast genome sequence of the CAM epiphyte Spanish moss (*Tillandsia usneoides*, Bromeliaceae) and its comparative analysis

**DOI:** 10.1371/journal.pone.0187199

**Published:** 2017-11-02

**Authors:** Péter Poczai, Jaakko Hyvönen

**Affiliations:** 1 Finnish Museum of Natural History (Botany), University of Helsinki, Helsinki, Finland; 2 Dept. Biosci. (Plant Biology), University of Helsinki, Helsinki, Finland; Austrian Federal Research Centre for Forests BFW, AUSTRIA

## Abstract

Spanish moss (*Tillandsia usneoides*) is an epiphytic bromeliad widely distributed throughout tropical and warm temperate America. This plant is highly adapted to extreme environmental conditions. Striking features of this species include specialized trichomes (scales) covering the surface of its shoots aiding the absorption of water and nutrients directly from the atmosphere and a specific photosynthesis using crassulacean acid metabolism (CAM). Here we report the plastid genome of Spanish moss and present the comparison of genome organization and sequence evolution within Poales. The plastome of Spanish moss has a quadripartite structure consisting of a large single copy (LSC, 87,439 bp), two inverted regions (IRa and IRb, 26,803 bp) and short single copy (SSC, 18,612 bp) region. The plastid genome had 37.2% GC content and 134 genes with 88 being unique protein-coding genes and 20 of these are duplicated in the IR, similar to other reported bromeliads. Our study shows that early diverging lineages of Poales do not have high substitution rates as compared to grasses, and plastid genomes of bromeliads show structural features considered to be ancestral in graminids. These include the loss of the introns in the *clp*P and *rpo*C1 genes and the complete loss or partial degradation of *acc*D and *ycf* genes in the Graminid clade. Further structural rearrangements appeared in the graminids lacking in Spanish moss, which include a 28-kb inversion between the *trn*G-UCC–*rps*14 region and 6-kb in the *trn*G-UCC–*psb*D, followed by a third <1kb inversion in the *trn*T sequence.

## Introduction

Chloroplasts, considered to have originated from cyanobacteria through endosymbiosis, are organelles responsible of photosynthesis to provide essential energy for plants and numerous other lineages of eukaryotes [1]. In angiosperms, chloroplast (cp) genomes exist in circular DNA forms [2] ranging from 120 to 160 kb in length [3]. Although, the extracted cpDNA is found in various structural forms, including branched-linear multigenomic molecules, unit-genome-sized linear isomers and head-to-tail concatemers, and less-than-genome-sized fragments [2]. Most plastid genomes have a quadripartite organization comprising two copies of 20 to 28 kb Inverted Repeats (IRs) which separate the rest of the genome into a 80–90 kb Large Single Copy region (LSC) and a 16–27 kb Small Single Copy region (SSC). In angiosperms, the plastid genome usually encodes four rRNAs, 30 tRNAs, and about 80 unique proteins. Advances in next-generation sequencing technologies [4,5] have resulted in a rapid increase in completed plastid genomes [6] sampled widely across green plants. The use of whole plastomes to infer phylogenies (i.e. phylogenomics) are increasing resolution and support values for relationships that have varied among, or been unresolved, in earlier single- and multi-gene studies [7].

**Fig 1:**
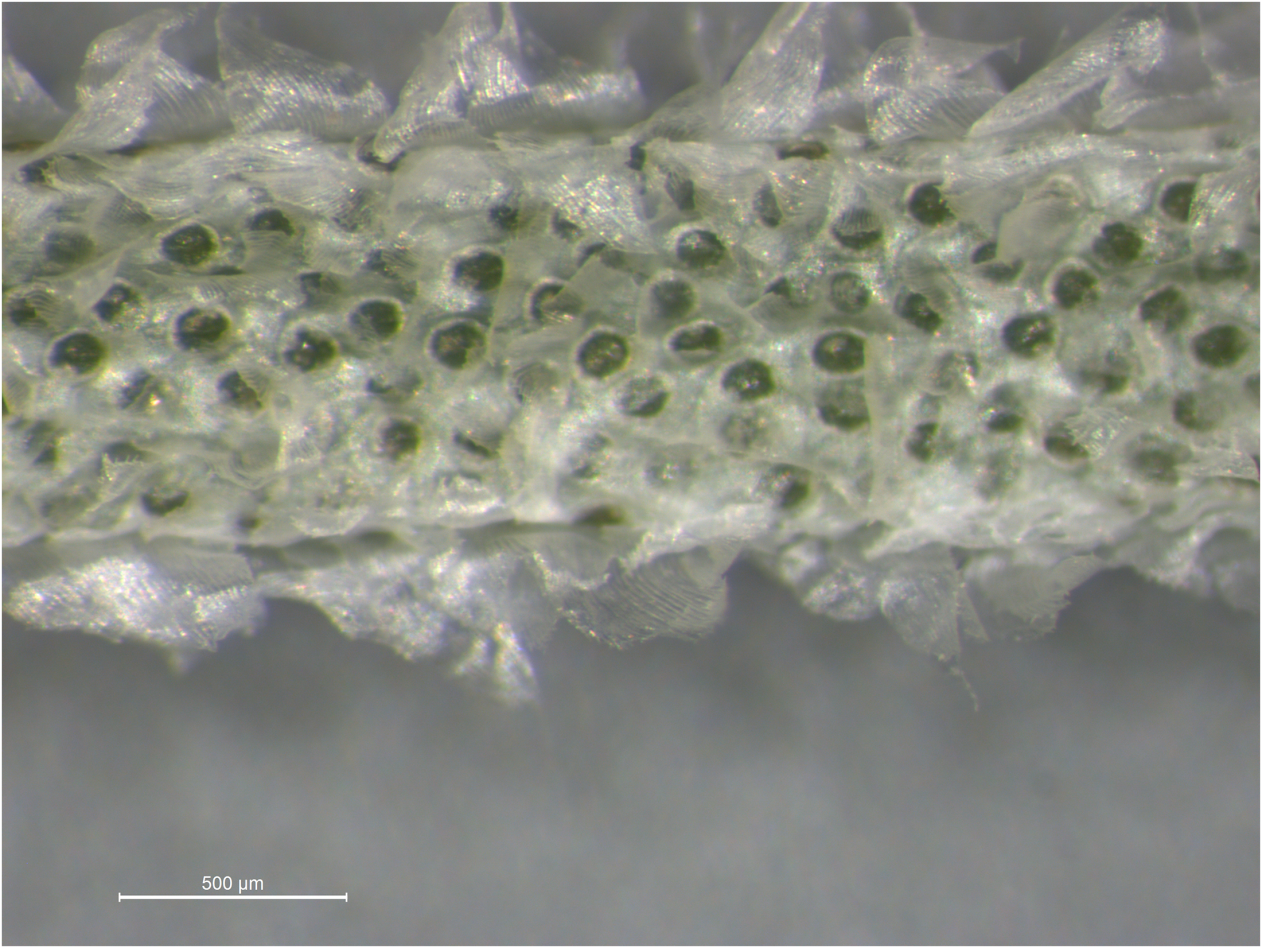
Close-up microscope photo of Spanish moss (*Tillandsia usneoides* L.). Peltate scales (trichomes) are displayed.

The family Bromeliaceae or the bromeliads (58 genera, ca. 3,140 species), constitute one of the most morphologically distinctive, ecologically diverse, and species-rich clades of flowering plants native to the tropical and warm temperate areas of the New World [8]. The family includes both epiphytes, such as Spanish moss (*Tillandsia usneoides* (L.) L.), and terrestrial species, like the pineapple (*Ananas comosus* (L.) Merr.). Many bromeliads are able to store water in a structure formed by their tightly-overlapping leaf bases. However, the family is diverse enough to include the tank bromeliads, grey-leaved epiphyte *Tillandsia* spp., and a large number of desert-dwelling succulents. Spanish moss belongs to subg. *Diaohoranthema* (Beer) Baker of the Tillandsioid core clade [9,10] with autogamous or cleistogamous flowers characterized by polyembryony [11]. Species of *Tillandsia*, like many others of the family Bromeliaceae, rely on foliage for water absorption via epidermal trichomes (Fig 1) for absorbing atmospheric water, mineral and organic nutrients. These structures have thus partially replaced functions of roots. This represents an advantage in habitats where root absorption is not effective, such as on trees and rocks, although the reduced root systems usually have retained a role as an anchor [12].

**Fig 2:**
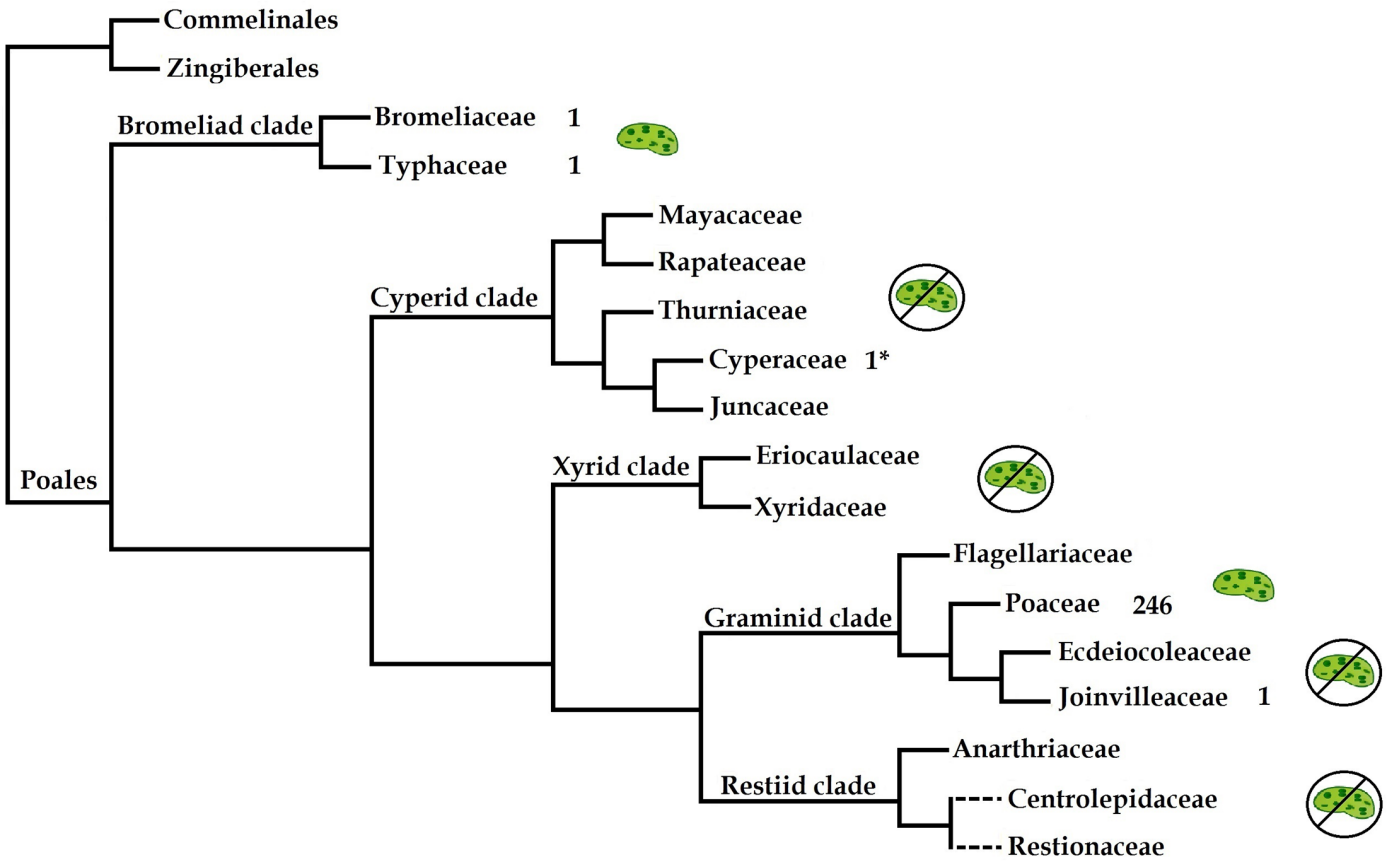
Phylogenetic tree showing the major clades and families of Poales, simplified from APG IV [16]. Dashed lines indicate families with uncertain position. Numbers indicate the available plastid genome sequences in NCBI Organelle Genome Resources (searched on 8.11.2016). Asterisk in case of Cyperaceae indicate the presence of one available (*Carex siderosticta*, NC027250) but currently unpublished sequence.

The predominantly wind-pollinated order Poales includes about one third of all monocot species, with c. 20,000 species dominating modern savanna and steppe vegetation [13]. The grasses not only include many economically important crops such as rice (*Oryza sativa* L.), maize (*Zea mays* L.) and wheat (*Triticum aestivum* L.) but also dominate various natural and agricultural landscapes of the world [14]. With the accomplished and ongoing genome sequencing of many cereals, grasses have become a model system for functional and comparative genomic research [15]. Recent improvements in understanding relationships within the order have successfully incorporated the phylogenomic analysis of complete chloroplast genome sequences to increase resolution in poorly supported nodes of the Poales phylogenetic tree [5,7]. In the current Angiosperm Phylogeny Group (APG) IV [16] scheme Poales is divided to five major clades (Fig 2). The majority of chloroplast phylogenomic studies have been concentrated on the Graminid clade and more specifically on Poaceae.

Up to date no plastid genome sequences have been available of the Xyrid and Restiid clades while such information is also sparse in the Bromeliad and Cyperid clades. The Bromeliad clade is a non-grass basal lineage, by adding further sequence information from this group to the phylogenetic tree of Poales we could gain insight to unique changes in genome organization of deeper clades and groups in the economically important Graminid clade. In this study, we report the assembly, annotation and structural analysis of the complete plastid genome of *Tilalndsia usneoides* of the Bromeliad clade and we compare its organization (gene content, IR expansion/contraction, and structural rearrangement) with nine plastid genomes of Poales. This comparative analysis was performed to gain further insights into the overall evolutionary dynamics of the plastid genomes in the Bromeliad clade and to better determine phylogenetic relationships in Poales.

## Materials and methods

### Chloroplast isolation

Spanish moss plant material was collected in the greenhouse of the Kaisaniemi Botanical Garden, University of Helsinki, Finland (60.1753; 24.9460). Our study did not require a field permit. The plant samples were obtained from *Index Seminum* seed exchange, and the specimen is curated by the Finnish Museum of Natural History. *Tillandsia usneoides* is not listed under the IUCN red list nor by CITES. Chloroplasts were isolated from 20 g of fresh leaves according to the modified high salt method of Shi et al. [17]. After the final centrifugation step DNA was extracted using the DNEasy Plant Mini Kit (Qiagen) following the manufacturer's instructions. The DNA concentration was quantified by using a Quant-iT dsDNA HS assay kit and a Qubit fluorometer (Invitrogen). The extracted DNA was also evaluated on 0.8% agarose gels by electrophoresis.

### Multiply-primed rolling circle amplification

Multiply-primed rolling circle amplification (RCA) was used to produce an abundance of purified chloroplast DNA template in preparation for sequencing following the method described by Atherton et al. [18]. The technique involves isothermal strand-displacing amplification using multiple primers and is capable of yielding a large amount of product from very little starting DNA template [19]. Phi29, the DNA polymerase used in multiply-primed RCA, is reported to have a very low level of amplification bias making the template suitable for whole genome sequencing [20]. Extracted DNA was amplified in this way using a REPLI-g Mini Kit (Qiagen) following the manufacturer's instructions, with the exception that samples were incubated at room temperature for 9 min rather than the recommended 3 min. This extension time consistently produced better results with different plant samples. The kit produced ~5 μg of product for each sample.

### DNA sequencing

Illumina paired-end sequencing libraries, with average insert size of 300 bp were prepared using the TruSeq DNA Sample prep kit (Illumina, San Diego, CA, USA). To verify the size of fragments the template size distribution was checked by running on an Agilent Technologies 2100 Bioanalyzer using a DNA 1000 chip. The libraries were sequenced from both ends of the molecules to a total read length of 150 bp from each end using an Illumina MiSeq platform. The raw.bcl files were converted into demultiplexed compressed fastq files using Casava 1.8.2 (Illumina).

### Genome assembly

Raw reads were first filtered to obtain high-quality clean data by removing adaptor sequences and low quality reads with Q-value ≤ 20 using Trimmomatic [21]. Error correction, removal of duplicated raw reads, and removal of contaminated raw reads were conducted using BBTools [22]. Plastid reads were filtered by reference mapping to Poales plastid genome sequences using Geneious 9.1.7. [23] with medium-low sensitivity and 100 iterations. From the collected reads a *de novo* assembly was carried out with the built-in Geneious assembler platform with zero mismatches and gaps allowed among the reads. The similar procedure was conducted with Velvet v1.2.10 [24] with k-mer length 37, minimum contig length 74 and default settings by applying a 400× upper coverage limit. The resulting contigs were then circularized by matching end points. The results of the reference mapping and two *de novo* methods were compared and inspected. Sanger-based gap closure and IR junction verification was carried out following Moore et al. [25].

### Genome annotation

Plastomes were annotated using DOGMA [26], Geneious and tRNAscan-SE [27], with comparisons to all published plastid genomes of bromeliads and other Poales using manual adjustments and in house scripts. The exact positions of genes were further confirmed using local BLAST searches against representative plastomes of Poales deposited in the NCBI genome database. Annotations were translated in Geneious and examined by eye; problematic annotations were removed and further curated. The gene maps of the genomes were drawn by using OGDraw v1.2 [28].

### Repeat analysis

We used the online REPuter software [29] to identify and locate forward, palindrome, reverse, and complement repeat sequences with n ≥30 bp and a sequence identity ≥90%. We classified repeats larger than 10 bp into the following groups: (i) tandem repeats (T), (ii) direct repeats dispersed in the genome (D), (iii) repeats found in reverse orientation dispersed in the genome (R), (iv) palindromic repeats forming hairpin loops in their structure (P) and (v) repeats found in reverse complement orientation. To assess the number of repeats in other chloroplast genomes, we ran the same REPuter analyses against the chloroplast genomes of the other nine species of Poales that were used in the comparative analyses. Folding structures of putative palindromic repeats were visualized with the implemented feature of Geneious. Simple sequence repeats (SSRs) were identified using MISA [30]. We applied a threshold seven to mononucleotide repeats, four to dinucleotide repeats and three to tri-, tetra, penta-, and hexanucleotide repeats. MISA allowed the identification and localization of perfect microsatellites as well as compound microsatellites which were set to be interrupted by 100 bp. The output file of MISA was manually rewritten into GFF3 format and exported to Geneious to compare the distribution of SSRs with other related plastid genomes.

### Genome analyses

Relative synonymous codon usage (RSCU) was calculated on the basis of protein-coding and tRNA genes using MEGA6.06 [31]. RSCU is defined as the ratio of the observed frequency of codons to the expected frequency given that all the synonymous codons for the same amino acids are used equally [32]. RSCU values have no relation to the amino acids usage and the abundance ratio of synonymous codons, which can directly reflect the bias of synonymous codon usage [33]. The number of base substitutions per site from averaging over all 30 sequence pairs were calculated using the Kimura 2-parameter model. Standard error estimate(s) were obtained by a bootstrap procedure (500 replicates). Codon positions included were 1st+2nd+3rd+Noncoding. All positions containing gaps and missing data were eliminated.

Gene content comparisons were performed using Multipipmaker [34]. Comparisons involved nine Poales plastomes (S1 Table) and one outgroup species *Zingiber spectabile* Griff. (Zingiberaceae). The comparison included *Tillandsia usneoides* (current study), *Ananas comosus* from Bromeliaceae and *Typha latifolia* L., all three belonging to the Bromeliad clade, and nine grasses: *Bambusa bambos* (L.) Voss., *Deschampsia antarctica* E. Desv., *Musa textilis* Née, *Triticum aestivum*, *Zea mays* and *Zizania aquatica* L. *T*. *useneoides* was used as the reference genome by including an exon file in the analysis. Gene orders were examined by pair-wise comparisons between all ten genomes using PipMaker [35].

Codon-based Z test of selection (neutral, positive, purifying) averaging over all selected sequence pairs were conducted using MEGA6.06. The dN/dS value quantifies selection pressures by comparing the rate of substitutions at silent or synonymous sites (dS), which are presumed to be neutral, to the rate of substitutions at non-silent or non- synonymous sites (dN), which possibly experience selection [36]. The ratio is often used as a measure of selective pressure indicating neutral substitution rates (dN/dS = 1), positive (dN/dS > 1) and purifying selection (dN/dS < 1), respectively. The variance of the difference was computed using the bootstrap method (1,000 replicates). Analyses were conducted using the Nei-Gojobori method. Gene groups were categorized according to gene function or subunits that form a functional complex; values were, respectively, combined for *atp*-, *ndh*-, *pet-*, *psa*-, *psb*-,*rpl*-, *rps*-, and *rpo*-genes. We compared the results obtained by Codon-based Z tests by repeating the positive selection tests with HyPhy [37] using the Datamonkey [38] interface for the *rps* genes using a HKY85 substitution model. We have run FUBAR [39] to investigate negative selection, BUSTED [40] to underline the presence of diversifying selection and MEME [41] to find site-specific episodic of diversifying selection. Since BUSTED [40] could be used to test both site-level and branch-level in gene-wild selection, the results of BUSTED [40] in site-level were compared with those from FUBAR [39], MEME [41]. In order to generate robust results we considered sites and branches under diversifying positive selection only if they were confirmed by two methods. We also used the program RELAX implemented in HyPhy to distinguish positive from relaxed selection [37] using all platid genes from 30 Poales genomes.

### Taxon sampling for phylogenomic analyses

Poales are a large order of angiosperms consisting of five major clades and sixteen families (APG IV [16]). For our study we utilized the plastome sequences currently available in NCBI database (8.11.2016). The database is biased containing 246 plastid genomes from Poaceae while no genomes are available from the Xyrid and Restiid clades. The aim of our study is to compare the structure of *Tillandsia usneoides* plastome with those of the other Poales and investigate the phylogenetic position of the Bromeliad clade. For this reason we systematically sampled all clades of the highly diverse Poaceae by including taxa from all twelve clades.

### Phylogenetic analyses

For phylogenetic analyses 80 genes were selected from complete chloroplast genome sequences. Homologous regions were extracted based on genome annotation features, which were refined and edited using MAFFT [42] alignments and BLAST searches. Selected regions were separately aligned with MAFFT and concatenated in a final fasta file (S1 and S2 Files). Altogether 30 plastid genomes (S1 Table) were included in our matrix with 27 genomes of Poales and those of *Musa textilis* and *Curcuma flaviflora* S. Q. Tong plus *Zingiber spectabile* used as outgroup terminals to root the resulting trees. Initially, after MAFFT alignment matrices included 86,065 characters. Maximum–likelihood (ML) analyses were run using RAxML v8.0 [43]. The data matrix was partitioned by gene (n = 80) and allowed separate base frequencies, α-shape parameters, and GTR evolutionary rates to be estimated for each. The best scoring ML tree was calculated under GTR-GAMMA after running jModelTest2 [44]. Branch support values were obtained from 10,000 non-parametric bootstrapping.

We performed parsimony analyses on this same matrix using Nona [45] within the WinClada shell [46]. Prior to these analyses we used the WinClada command “Mop uninformative characters” to exclude parsimony uninformative characters. This resulted in a matrix with 15,687 characters. Nona analyses were performed using processor time as a seed to randomize the order of the terminals with the following settings: hold 30,000 (holding a defined number of trees), 100 replications (search performed with multiple tree bisection–reconnection algorithm mult*max*), and hold/3 (to define the starting trees for each replication). This was supplemented by another search with 1,000 replications and the number of starting trees defined as 20. We used the same programs to calculate bootstrap support values. These values were obtained with 1,000 replications of ten search replications (mult*10) and with one starting tree per replication (hold/1). As with the actual search of the parsimonious trees these analyses used processor time as a seed for randomization, and tree bisection-reconnection algorithm was used (max*).

## Results and discussion

### Genome assembly and plastome structure

**Fig 3:**
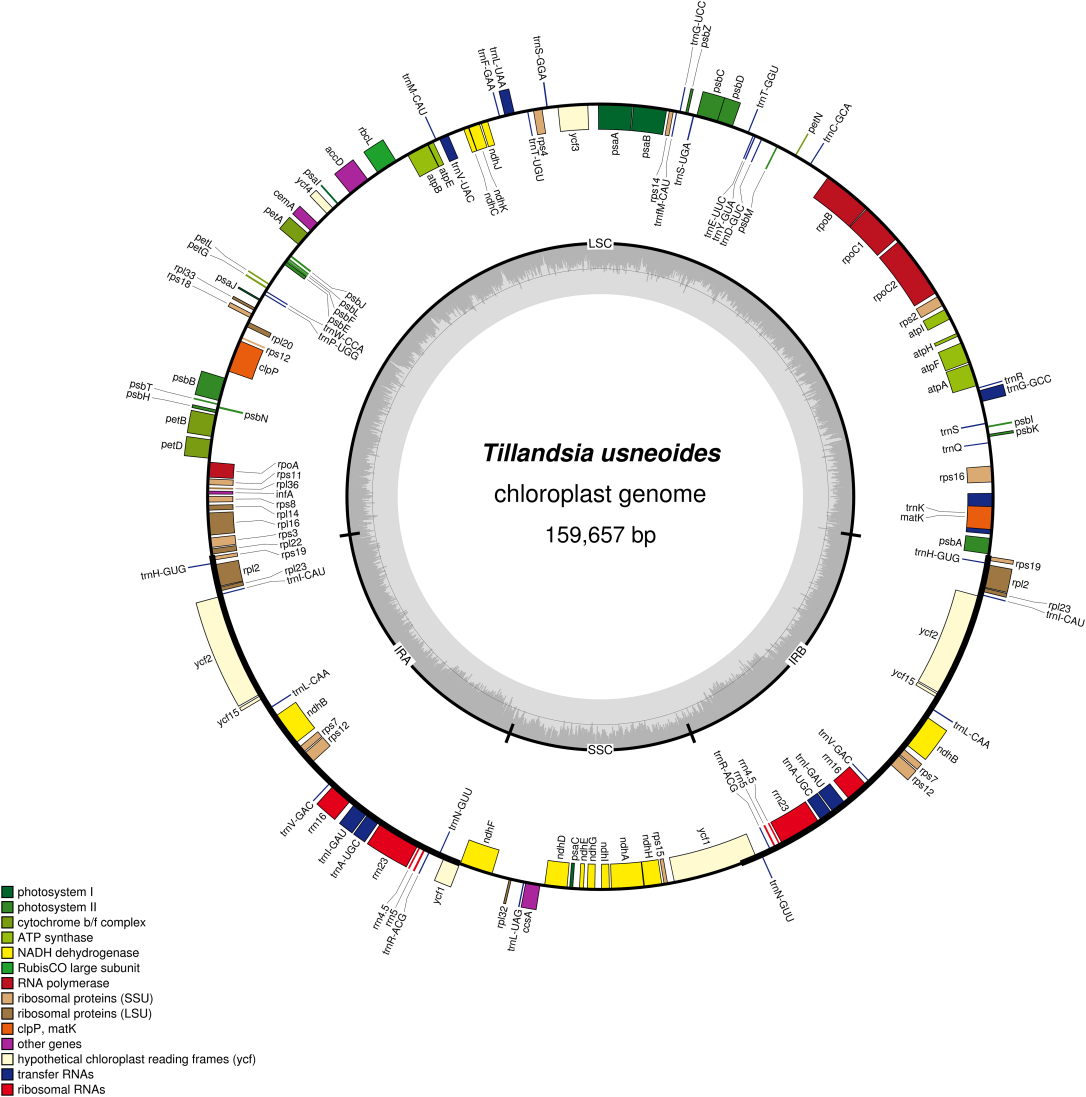
The circular representation of the plastid genome of Spanish moss (*Tillandisa usneoides*). Structural organization of the gene content ring was color coded based on its functional category. The innermost circle denotes the GC content across the genome. The genes that were transcribed counter-clockwise and clockwise were at the outer and inner ring, respectively. The illustration was drawn using OGDRAW.

We sequenced the plastid genome of Spanish moss using the Illumina genome analyzer platform. Illumina paired-end (2 × 150 bp) sequencing produced a total of 3,416,442 paired-end reads, with an average fragment length of 277 bp. 2,865,232 paird-end reads were retained after quality filtering. Low quality reads (Q20) were filtered out, and the remaining high quality reads (2,865,232) were mapped to the *Ananas comosus* plastid genome (NC026220) to collect chloroplast specific reads (1,211,499). Unmapped reads were discarded as mitochondrial and/or nuclear genomic contamination and were not used in our study. For genome assembly we used one reference mapping and two *de novo* methods. Filtered reads mapped to the pineapple reference genome resulted in an entire contig showing good agreement with published Poales genome sequences. Based on these collected reads we used Geneious and Velvet to produce a single contiguous fragment representing the plastid genome. The three assemblies were compared and discrepancies were manually resolved. The final assembly had an average 1088× genome coverage. To validate the assembly, four junction regions between the IRs and SSC/LSC were confirmed by PCR amplifications and Sanger sequencing (S2 Table). We compared the sequenced results with the assembled genome directly and no mismatch or indel was observed supporting the accuracy of our assembly. After annotation this genome sequence was submitted to GenBank (ID: KY293680). The complete chloroplast genome of *Tillandsia usneioides* is 159,657 bp in length and includes the typical quadripartite angiosperm plastome structure and arrangement (Fig 3). The genome is consisted of a large single copy (LSC, 87,439 bp), inverted region A (IRa, 26,803 bp), short single copy (SSC, 18,612 bp) and inverted region B (IRb, 26,803 bp). Overall the Spanish moss plastid genome had a GC content of 37.2%, consistent with other reported bromeliad plastid genomes [47].

However, both inverted regions had a higher GC content of 42.7%, whereas the GC content for LSC and SSC were 37.2% and 31.2%, respectively. The genome contained 134 genes with 88 being unique protein-coding genes and 20 of these are duplicated in the IR including *rps*12, *rps*7, *ndh*B, *rpl*23, *rpl*2 and *rps*19 (Table 1).

**Table 1:** List of genes in the chloroplast genome of Spanish moss.

	Group of genes	Name of genes
Protein synthesis and DNA-replication	Transfer RNAs	*trn*A-UGC^b^ (×2), *trn*C-GCA, *trn*D-GUC, *trn*E-UUC, *trn*F-GAA, *trn*fM-CAU, *trn*G-GCC^b^, *trn*G-UCC, *trn*H-GUG (×2), *trn*I-CAU (×2), *trn*I-GAU^b^ (×2), *trn*K-UUU, *trn*L-CAA (×2), *trn*L-UAA^b^, *trn*L-UAG (×2), *trn*M-CAU, *trn*N-GUU (×2), *trn*P-UGG, *trn*Q-UUG, *trn*R-ACG (×2), *trn*R-UCU, *trn*S-GCU, *trn*S-GGA, *trn*S-UGA, *trn*T-GGU, *trn*T-UGU, *trn*V-GAC (×2), *trn*V-UAC^b^, *trn*W-CCA, *trn*Y-GUA
	Ribosomal RNAs	*rrn*16 (×2), *rrn*23 (×2), *rrn*4.5 (×2), *rrn*5 (×2)
	Ribosomal protein small subunit	*rps*2, *rps*3, *rps*4, *rps*7 (×2), *rps*8, *rps*11, *rps*12^c^ (×2), *rps*14, *rps*15, *rps*16, *rps*18, *rps*19 (×2)
	Ribosomal protein large subunit	*rpl*2^b^ (×2), *rpl*14, *rpl*16^b^, *rpl*20, *rpl*22, *rpl*23 (×2), *rpl*32, *rpl*33, *rpl*36
	Subunits of of RNA polymerase	*rpo*A, *rpo*B, *rpo*C1^b^, *rpo*C2
Photosynthesis	Photosystem I	*psa*A, *psa*B, *psa*C, *psa*I, *psa*J
	Photosystem II	*psb*A, *psb*B, *psb*C, *psb*D, *psb*E, *psb*F, *psb*H, *psb*I, *psb*J, *psb*K, *psb*L, *psb*M, *psb*N, *psb*T, *psb*Z
	Cythochrome b/f complex	*pet*A, *pet*B^b^, *pet*D^b^, *pet*G, *pet*L, *pet*N
	ATP synthase	*atp*A, *atp*B, *atp*E, *atp*F^b^, *atp*H, *atp*I
	NADH-dehydrogenase	*ndh*A^b^, *ndh*B^b^ (×2), *ndh*C, *ndh*D, *ndh*E, *ndh*F, *ndh*G, *ndh*H, *ndh*I, *ndh*J, *ndh*K
	Large subunit Rubisco	*rbc*L
Miscellaneous group	Translation initiation factor IF-1	*inf*A
	Acetyl-CoA carboxylase	*acc*D
	Cytochrome c biogenesis	*ccs*A
	Maturase	*mat*K
	ATP-dependent protease	*clp*P^a^
	Inner membrane protein	*cem*A
Pseudogene unknown function	Conserved hypothetical chloroplast ORF	*ycf*1 (×2), *ycf*2 (×2), *ycf*3^a^, *ycf*4, *ycf*15 (×2)

^a^Gene containing two introns;

^b^Gene containing a single intron;

^c^Gene divided into two independent transcription units

Additionally, 38 unique tRNA genes representing all the 20 amino acids are distributed throughout the genome (one in the SSC region, 21 in the LSC and eight repeated in each IR region). Four rRNA genes are also identified in the plastid genome and these are duplicated in the IR regions.

Of the 134 genes, 16 have a single intron (10 protein coding genes and 6 tRNA genes) and two genes (*clp*P and *ycf*3) have two introns (all are protein coding) (Table 2). Out of the 17 genes with introns, 13 are located in the LSC (9 protein-coding and 3 tRNAs), one is located in the SSC (*ndh*A) and four in the IR region (2 protein-coding and 2 tRNAs).

**Table 2:** The genes having intron in the *Tillandsia usneoides* plastid genome and the length of the exons and introns.

Gene	Location	Exon1 (bp)	Intron1 (bp)	Exon2 (bp)	Intron2 (bp)	Exon3 (bp)
*rps*16	LSC	212	826	42		
*trn*G-GCC	LSC	23	687	48		
*atp*F	LSC	411	834	144		
*rpo*C1	LSC	1626	706	435		
*ycf*3	LSC	153	747	228	726	132
*trn*L-UAA	LSC	35	576	50		
*trn*V-UAC	LSC	35	586	39		
*rps*12*	LSC	114		26	541	232
*clp*P	LSC	252	669	291	879	69
*pet*B	LSC	6	783	642		
*pet*D	LSC	8	745	474		
*rpl*16	LSC	402	1076	9		
*rpl*2	IR	432	666	393		
*ndh*B	IR	756	700	777		
*trn*I-GAU	IR	37	945	35		
*trn*A-UGC	IR	38	801	35		
*ndh*A	SSC	540	1061	549		

*rps12 is a trans-spliced gene with 5' end exon located in the LSC region and the duplicated 3'end exon located in the IR regions.

One gene, *rps*12 was found to be trans-spliced with the 5’ end exon located in the LSC region and the duplicated 3’ end exon located in the IR region. We also observed four cases of overlapping genes, namely *psb*D/*psb*C, *ndh*K/*ndh*C, *atp*E/*atp*B, and *ycf*1/*ndh*F.

The *ycf*15 gene contains three internal stop codons, indicating that this gene is nonfunctional pseudogene. This was caused by a single base change and deletion of five bases causing a frame-shift in the 3’ end. This has been reported also in pineapple [47] and can be observed in *Typha latifolia*, however, as unannotated [48]. The gene has a GTG start codon common to most chloroplast genomes where *ycf*15 is found in contrast to have the ATG codon in *Camellia* L. where this gene is intact [49]. The *ycf*15 is a gene of uncertain origin. It does not possess orthologues in eubacteria and its protein-coding validity is also questionable [49]. It has been disabled in many angiosperm lineages, for example monocots, most rosids and it has also been lost in some groups i.e. *Illicium* L. and *Acorus* L. Recent studies [49] have not been able to show whether this gene is able encode a protein or how it has evolved in angiosperms.

Sequence analysis indicates 58.66%, 6.77%, and 5.66% of the genome sequences to encode proteins, tRNAs and rRNAs, while the remaining 28.89% are non-coding and comprises of intergenic spacers and pseudogenes. All the tRNAs required for the synthesis of the protein coding genes were identified in the Spanish moss plastid genome, and this is consistent with other angiosperm chloroplast genomes. The 88 protein-coding genes represent 93,664 nucleotides coding for 31,221 codons. On the basis of protein-coding and tRNA gene sequences the frequency of RSCU was calculated (Table 3). The most common amino acid is lysine (K), with 1,243 (3.98%) while the least encoded one is alanine (109, 0.34%). The codon usage is biased towards a high representation of A and T at the third codon position as reported for angiosperms plastid genomes [50].

**Table 3:** Relative synonymous codon usage (RSCU) of *Tillandsia usneoides* is given in parentheses following the codon frequency.

Codon	Count	RSCU	Codon	Count	RSCU	Codon	Count	RSCU	Codon	Count	RSCU
UUU(F)	1205	1.22	UCU(S)	581	1.3	UAU(Y)	951	1.36	UGU(C)	456	1.12
UUC(F)	763	0.78	UCC(S)	422	0.95	UAC(Y)	443	0.64	UGC(C)	356	0.88
UUA(L)	751	1.43	UCA(S)	611	1.37	UAA(*)	434	0.91	UGA(*)	579	1.21
UUG(L)	675	1.29	UCG(S)	335	0.75	UAG(*)	425	0.89	UGG(W)	611	1
CUU(L)	615	1.17	CCU(P)	340	1.23	CAU(H)	529	1.37	CGU(R)	263	0.72
CUC(L)	337	0.64	CCC(P)	221	0.8	CAC(H)	243	0.63	CGC(R)	135	0.37
CUA(L)	479	0.91	CCA(P)	368	1.33	CAA(Q)	652	1.37	CGA(R)	356	0.97
CUG(L)	285	0.54	CCG(P)	180	0.65	CAG(Q)	302	0.63	CGG(R)	241	0.66
AUU(I)	1100	1.26	ACU(T)	385	1.15	AAU(N)	1052	1.33	AGU(S)	420	0.94
AUC(I)	704	0.81	ACC(T)	293	0.87	AAC(N)	527	0.67	AGC(S)	303	0.68
AUA(I)	805	0.93	ACA(T)	457	1.36	AAA(K)	1243	1.34	AGA(R)	752	2.05
AUG(M)	683	1	ACG(T)	209	0.62	AAG(K)	608	0.66	AGG(R)	449	1.23
GUU(V)	531	1.43	GCU(A)	252	1.26	GAU(D)	714	1.49	GGU(G)	388	1
GUC(V)	229	0.62	GCC(A)	160	0.8	GAC(D)	247	0.51	GGC(G)	188	0.48
GUA(V)	466	1.25	GCA(A)	276	1.39	GAA(E)	878	1.37	GGA(G)	598	1.54
GUG(V)	263	0.71	GCG(A)	109	0.55	GAG(E)	406	0.63	GGG(G)	382	0.98

*translation termination (stop codon)

### Repetitive sequences in the chloroplast genome

Repeated stretches of sequence may play a role in the rearrangement of plastid genomes and generating divergent regions via illegitimate recombination and slipped-strand mispairing [51–53]. For example the analysis of repeat sequences demonstrated that tandem repeats had an important impact on sequence diversification between legume plastomes [54]. In our analysis we first concentrated to locate chloroplast simple sequence repeats (cpSSRs), which are generally short mononucleotide tandem repeats and are commonly found in the noncoding regions of the plastid genomes. Using MISA we identified and located 432 SSRs in the Spanish moss plastome, of which 281 were mono-, 67 di-, 61 tri-, 14 tetra- and two were pentanucleotides (S3 Table). From the total number of SSRs 147 occurred in compound formation that was made up of several combinations of SSRs interrupted by maximum distances of 100 bp. Poly-A/T stretches of variable size were the most abundant motifs found among SSRs with 61.57%. The complete list of identified SSRs is available in the S3 File. SSRs were more abundant in the LSC and SSC regions compared to IRs. From the total number of SSRs found in the *Tillandsia usneoides* plastome, 172 were found in coding regions of 45 genes. The most variable genes were *ycf*1/2, *ndh*A, *clp*P and *rpo*C1/C2 containing six to 14 SSRs. In a second step we identified 49 repeat structures using REPuter with a minimum size of 20 bp. Because REPuter overestimates the number of repeats thus we manually inspected the output file by locating the repeats using Geneious. We excluded many redundant repeats, which were entirely contained within other repeats. REPuter also identified repeating parts of duplicate tRNAs e.g. *trn*G-GCC and *trn*G-UCC (22 bp from 10,727 and 38,546) or *trn*S-UGA and *trn*S-GGA (20 bp from 37,648 and 47,125). Gene similarity and the repeats found among tRNAs provided evidence of possible gene duplications, also shared among most embryophytes. After the manual pruning process, we located 25 repeat sequences in the Spanish moss plastid genome, consisting of two reversed, two complementary, three direct, six tandem, nine palindromic and three mixed direct/palindromic repeats (Table 4). The largest repeat with a size of 126 bp was a tandem repeat found in the IGS region of *trn*D-GUC and *trn*Y-GUA. The majority of the repeats were palindromic distributed in intergenic spacer regions of the long single copy (LSC) region.

**Table 4:** Repeat sequences of *Tillandsia usenoides* chloroplast genome.

No	Type	Location		Region	Repeat unit	Period size (bp)	Copy number
1	T	*clp*P	intron	LSC	AGTAATAGTAGGTATAA	17	4
2	T	*psb*E—*pet*L	IGS	LSC	TAATAATAATAAATAAAAAA	20	2
3	T	*pet*D	intron	LSC	TTATATGGGTTTATTTCTGTTAT	23	2
4	T	*trn*D-GUC—*trn*Y-GUA	IGS	LSC	ATTTTACGGCCAAAAGTGGATAATCCATCTTTCAAT GAAAAAAAAAATTGATCCTTTCTCTTT	63	2
5	D	*rbc*L—*acc*D	IGS	LSC	AAAAAAAAGAGAATCATTTCT	21	2
6	D	*trn*S-GCU—*trn*G-GCC	IGS	LSC	TAAGTACTGGCCGGGACATTTCTTCTTTTATTCCATTGATTAT	42	2
7	D	*trn*S-GAA—*rps*4	IGS	LSC	TGCGGATGGATTAAGGCCCTTAGATCTATTTAGTTCGGCGAAA	42	2
8	C	*trn*S-GCU—*trn*G-GCC; *psb*E—*pet*L	IGS	LSC	TAATTATTATTATTTATTT	19	2
9	C	*psb*E—*pet*L; *clp*P	IGS/intron	LSC	TAGAAAAAAAAAAAAAACA	19	2
10	R	*ndh*C	CDS	LSC	ATCAAAAACA	10	2
11	P	*acc*D—*psa*I	IGS	LSC	TTCCAAATAAT	11	2
12	P	*trn*G-UCC—*trnf*M-CAU	IGS	LSC	TACTAACTACTA	13	2
13	P	*atp*I; *psb*A—*ycf*3	CDS/IGS	LSC	ATTTTCCAAGGTAAAAGG	18	2
14	P	*psb*T—*psb*N	IGS	LSC	TGAAGTAATGAGCCTCCC	18	2
15	P	*pet*N—*psb*M	IGS	LSC	AAAATGTGGTAGAAAGGACTATA	24	2
16	T	*ndh*F—*rpl*32	IGS	SSC	TCGGAAATCTTATGATACTCCT	22	2
17	R	*ndh*G—*ndh*I	IGS	SSC	GAAAGAAAAAAAAA	14	2
18	P	*ndh*G—*ndh*I	IGS	SSC	TCACTAAAAATA	12	2
19	P	*ndh*F -*rpl*32	IGS	SSC	TATATGTGATATA	13	2
20	P	*ccs*A	CDS	SSC	AAAGAAGTGTTTTT	14	2
21	P	*ycf*1	CDS	SSC	TTTGACTTTTATTTTTA	17	2
22	T	*ycf*2	CDS	IRa/IRb	GATATCGATATTGATGATAGTGACGATA	28	2
23	D/P	*ycf*2	IGS	IRa/IRb	CTTTTTGTCCAAGTCAC	17	2
24	P/D	*rrn*16—*trn*I-GAU	IGS	IRa/IRb	TTTTACGTCCCCATGTCG	18	2
25	D/P	*ycf*3 and *rps*12—*trn*V-GAC	intron/IGS	LSC/IRA/IRB	TACAGAACCGTACATGAGATTTTC	24	3

Abbreviations T = Tandem, D = direct, P = Palindromic, R = Reversed, C = Complementary, IGS = intergenic spacer, CDS = Coding Sequence, LSC = long single copy region, SSC = small single copy region, IR = inverted repeat

A small portion of repeats were found among nine coding regions in the CDS of *atp*I, *ndh*C, *ccs*A, *ycf*1, and *ycf*2, or in the intron of *clp*P, *pet*D and *ycf*3. Among the most variable genes were *clp*P and *ycf*2 containing two repeats while the *psb*E-*pet*L appeared to be the most variable containing three. We found no repeats in the *rpo*C2 gene, which is highly variable in Poales [55, 56]. The plastid genome of pineapple also, from Bromeliaceae, is in this respect similar having only single repeat in *rpo*C2 [47]. It seems that this region is highly variable exclusively in Poaceae. The dispersed repeats identified in *Tillandsia usneoides* could provide a basis for the development of markers for phylogenetic and population genetic studies.

### Protein-coding gene sequence diversity of Poales

To further explore the sequence diversity of the Spanish moss plastome we extracted 80 protein-coding genes from the alignment of Poales chloroplast genomes and computed the overall mean sequence distances (S4 Table). The results showed low levels of average sequence distance among Poales for protein-coding genes. Most of the genes (87.5%) had an average sequence distance of less than 0.10, and only 10 genes showed a value greater than 0.10. The ten most conserved genes were *ndh*B, *psb*L, *rps*12, *rpl*2, *rps*7, *psb*E, *psb*N, *pet*N, *pet*L and *psb*A (Table 5). Kim and Lee [57] showed that the majority of the *psa*, *psb* and *pet* gene classes show relatively slow evolutionary divergence. Contrary to the conserved nature of photosynthetic system the following genes were found to be the most divergent: *rpl*22, *rps*18, *ndh*F, *inf*A, *clp*P, *rpl*32, *mat*K, *ycf*1, *ycf*2 and *acc*D (Table 5). Two of these genes *ycf*2, *rpl*32 and *ndh*F, *ycf*1 were located in the IR and SSC regions while the remaining six divergent ones were all found in the LSC. Previous comparisons of chloroplast regions revealed differences among the most divergent regions of plastid genomes. Protein-coding genes such as *ycf*1, *ycf*2 *acc*D, *clp*P, *mat*K, *inf*A, *rpl*22 and *rpl*32 were observed to be the most divergent coding regions in vascular plastid genomes [58–60]. In most embryophyte lineages, *ycf*1 and *ycf*2 genes have elevated substitution rates and may have undergone pseudogenization [61]. The divergence of the *ycf* genes is not surprising as they have experienced many insertion/deletion events in Poales accounting for the reduction in chloroplast genome size among members of the Graminid clade [48]. Nuclear encoded and plastid targeted proteins similar to *ycf*1/*ycf*2 were not found in Poaceae lineages where both genes have been lost from the plastid genome [61].

**Table 5:** Estimates of average evolutionary divergence over 80 protein coding-gene sequences from Poales. Standard error estimate(s) are shown in the fourth column and were obtained by a bootstrap procedure (1000 replicates). The ten most divergent genes are shown in part A, while the ten most conserved genes are listed in part B.

	Functional Group	Gene	Location	d	S.E.
**A**	Acetyl-CoA carboxylase	*acc*D	LSC	0.297	0.065
	ATP-dependent protease	*clp*P	LSC	0.117	0.008
	Conserved hypothetical chloroplast ORF	*ycf*1	SSC	0.130	0.009
		*ycf*2	IR	0.204	0.068
	Maturase	*mat*K	LSC	0.119	0.005
	NADH-dehydrogenase	*ndh*F	SSC	0.104	0.004
	Ribosomal protein small subunit	*rps*18	LSC	0.105	0.011
	Ribosomal protein large subunit	*rpl*22	LSC	0.101	0.010
		*rpl*32	IR	0.119	0.015
	Translation initiation factor IF-1	*inf*A	LSC	0.115	0.016
**B**	Cytochrome b/f complex	*pet*N	LSC	0.033	0.010
		*pet*L	LSC	0.036	0.010
	NADH-dehydrogenase	*ndh*B	IR	0.014	0.001
	Photosystem II	*psb*A	LSC	0.038	0.003
		*psb*E	LSC	0.028	0.005
		*psb*L	LSC	0.014	0.005
		*psb*N	LSC	0.029	0.008
	Ribosomal protein large subunit	*rpl*2	IR	0.024	0.003
	Ribosomal protein small subunit	*rps*7	IR	0.028	0.004
		*rps*12	IR	0.018	0.002

The *mat*K and *ndh*F genes are well known to have high divergence and thus have been used in plant phylogenetic studies of various groups [62]. Most angiosperms appear to contain an intact chloroplast *inf*A gene, which codes for translation initiation factor 1. We also observed intact *inf*A genes in our alignment but sequences were prone to numerous SNPs accountable for the higher divergence of this gene. However, recent phylogenetic analyses show that chloroplast *inf*A has been lost repeatedly during angiosperm evolution and the gene has been transferred at least four times to the nuclear genome independently [58]. The divergence of the *acc*D was caused by partial deletion in several members of the Poales, which was also reported earlier [59].

### Comparison with other Poales

We selected ten chloroplast genomes for detailed comparison representing clades of Poales coupled with outgroup taxa (Fig 2). Our selection focused on general comparison of bromeliads with currently available Poales (accessed from GenBank 7.7.2016) rather than a detailed analyses of plastid genomes in the Graminid clade. In our comparison the size of the plastid genomes varied between 134,545 bp (*Triticum aestivum*) to 161,572 bp (*Typha latifolia*) (Table 6). Among the selected plastid genomes gene content and order were conserved. Epiphytic lifestyle seem not to be associated with any gene loss in *Tillandsia usneoides*. Bromeliads have maintained similar features of gene organizations with five major differences relative to the graminids.

**Table 6:** Comparison of major features of *Tillandsia usneoides* and nine commelinid plastid genomes.

	*Tillandsia*	*Ananas*	*Typha*	*Zingiber*	*Musa*	*Triticum*	*Deschampsia*	*Zizania*	*Bambusa*	*Zea*
GenBank Nr.	KY293680	NC026220	NC013823	NC020363	NC022926	NC002762	NC023533	NC026967	NC026957	NC001666
Size (bp)	159 657	159 636	161 572	155 890	161 347	134 545	135 362	136 354	142 772	140 384
LSC length (bp)	87 489	87 482	89 140	85 650	88 016	80 348	79 881	82 009	79 972	82 352
SSC length (bp)	18 612	18 622	19 652	18 444	18 989	12 791	12 519	12 587	12 868	12 536
IR length (bp)	26 803	26 766	26 390	25 898	27 171	20 703	21 481	20 879	24 966	22 748
Total Nr of genes	134	141	131	132	133	128	114	129	128	128
Nr of genes duplicated in the IR	20	24	18	21	20	18	12	18	19	18
Nr genes with introns	18	18	18	21	20	16	14	16	18	16
% GC content	37.2	37.4	33.8	36.3	35.9	37.2	38.3	39	37.2	37.4

**Fig 4:**
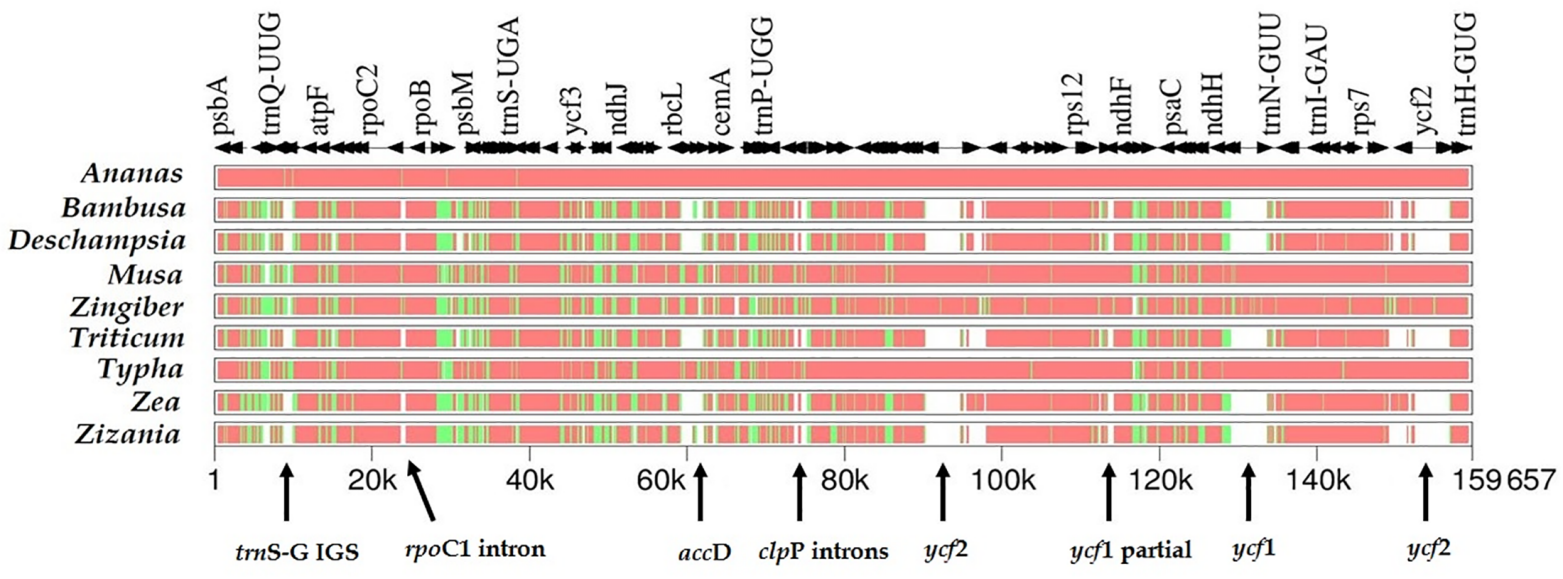
Multipip analysis showing overall sequence similarity of plastid genomes based on complete genome alignment. Levels of sequence similarity are indicated by red (75–100%), green (50–75%) and white (<50%). Comparison was made for ten plastid genomes using *Tillandsia usneoides* as the reference genome. Arrows indicate gene/intron losses and deletions; partial duplication of the ycf1 is due to IR expansion.

The *acc*D and *ycf*1/*ycf*2 genes were partially degraded (Fig 4), while the protein-coding genes *clp*P and *rpo*C1 have maintained their introns while they have been lost in graminids. We also found other differences among plastid genomes but the close inspection of aligned sequences indicated that they are due to annotation errors in the genomes submitted to GenBank.

**Fig 5:**
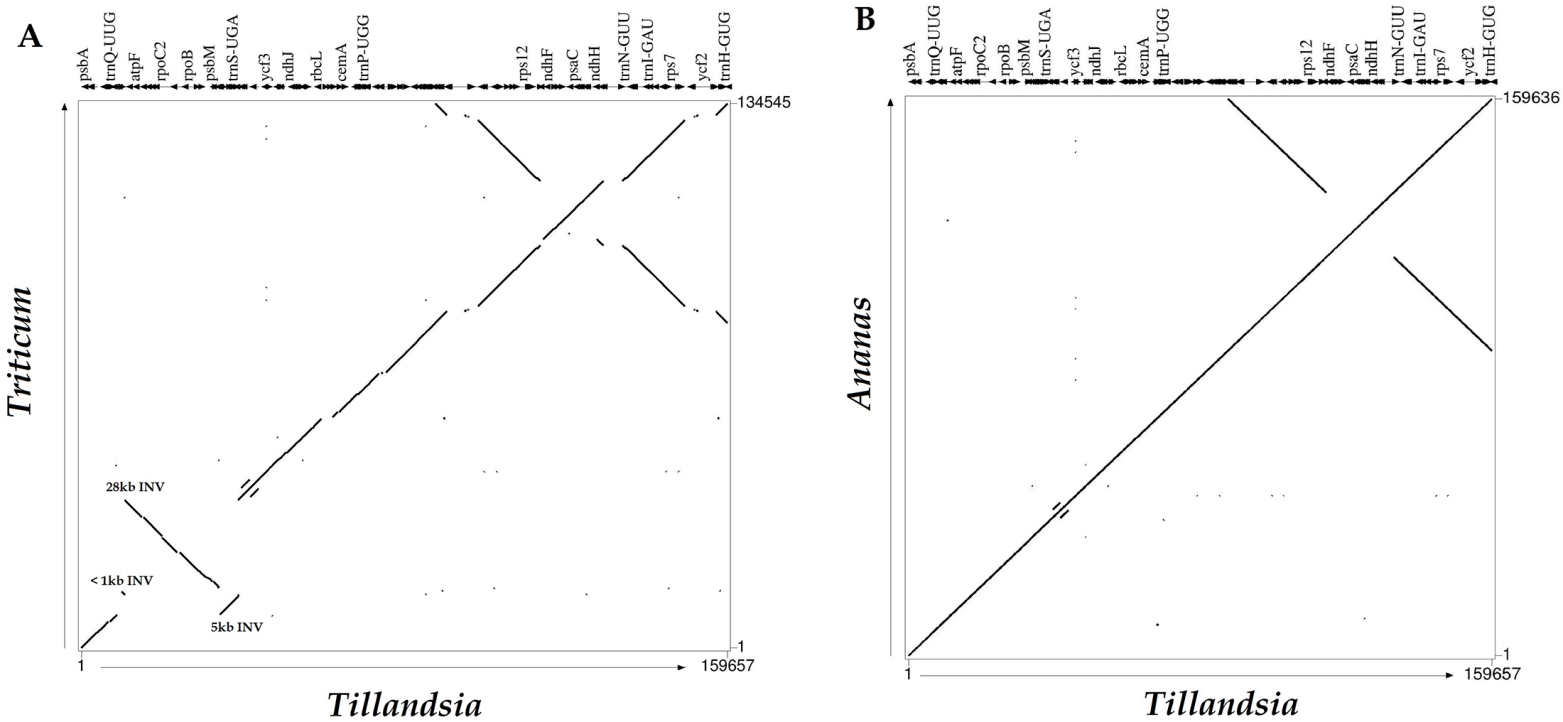
Percent identity plots. A. *Tillandsia usneoides* compared to *Triticum aestivum*, showing the three major inversions specific for grasses. B. *Ananas comosus* compared to *T*. *usneoides*.

The gene order between graminids and bromeliads is also characterized by three major inversions (Fig 5). The first is a larger 28-kb inversion between the *trn*G-UCC–*rps*14 region, while the second of six kb can be found in the *trn*G-UCC–*psb*D region.

The third, smallest inversion (< 1kb), is located in the *trn*T and flanking sequences. The second and third inversions are nested within the first 28-kb, while the two larger inversions are overlapping determining that the 28-kb inversion occurred prior of the smaller six kb inversion [61]. The 28-kb inversion in Poaceae might be a product of repeat-mediated intramolecular recombination as we found three repeats in the flanking regions. Another alternative explanation could be intramolecular recombination involving tRNA genes. Further studies focusing on clades of Poales with currently no plastid genome information available could assist in proposing hypotheses about the possible mechanisms giving rise to plastid genome inversions in grasses.

### Contraction and expansions of IRs

**Fig 6:**
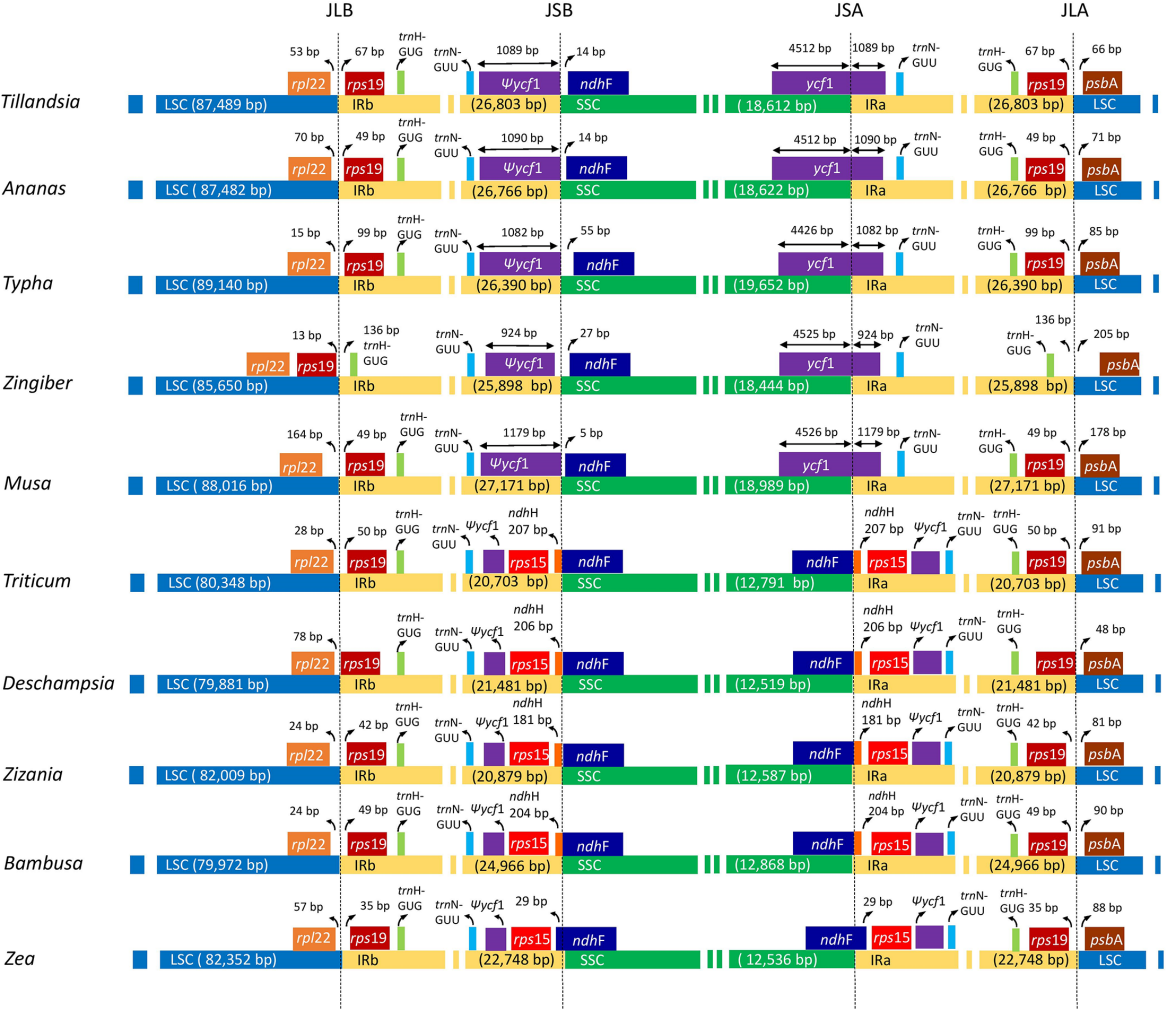
Comparison of plastid borders of LSC, SSC and IR regions among selected species of Poales. Selected genes or portions of genes are indicated by colored boxes. Gene and region lengths are not to scale (see Table 6).

Typically the IR of most plastid genomes is divided by four junctions JLB (IRb /LSC), JSB (IRb/SSC), JSA (SSC/IRa) and JLA (IRa/LSC). The size variation of angiosperm plastid genomes is primarily due to expansion and contraction of the IR and the SSC boundary regions [63]. Detailed comparison at the junction of the IR/SSC boundaries among the ten Poales plastid genomes are presented in Fig 6.

Despite the similar length of the IR regions in the ten species from 20,703 bp to 26,803 bp, some IR expansions and contractions were observed. The extent of the IR expansion to the intergenic spacer region between *rps*19 to *psb*A and *rps*19 to *rpl*22 varies from 13 to 205 bp. In all Poales plastid genomes examined the IR regions expanded to duplicate two genes the *trn*H-GUG and *rps*19 and their intergenic spacer consistent with previous reports [64–66]. The endpoint of this duplication seems to be conserved with 35–99 bp beyond the *rps*19 gene. In monocots the IRa/LSC is characteristically located downstream of the *psb*A while in eudicots for example in tobacco it is located upstream of *rps*19 and downstream of *trn*H-GUG [67]. The *psb*A-*trn*H-GUG region is commonly used as a DNA barcode including the highest percentage of variable sites among plastid markers [67]. However, there are serious problems with the alignment of barcodes due to variable IR expansion/contraction across genera or even different species of the same genus [67]. Previous studies showed that in some monocot lineages the *rps*19 gene copy located in the IRa is a truncated pseudogene and this duplication seem to have appeared in the early evolution of monocots [64]. At the IRb/SSC junction (JSB) the bromeliad clade shares synapomorphic structural features with outgroups including a truncated *ycf*1 pseudogene of 1082 to 1090 bp size. Contrary to this, members of the graminid clade (*Bambusa*, *Deschampsia*, *Triticum* and *Zizania*) have duplicate 181–207 bp of *ndh*H while *Zea* extends 29 bp of *nd*hF in the IR due to the truncation and scattered deletion of *ycf* genes.

### Phylogenetic relationships

**Fig 7:**
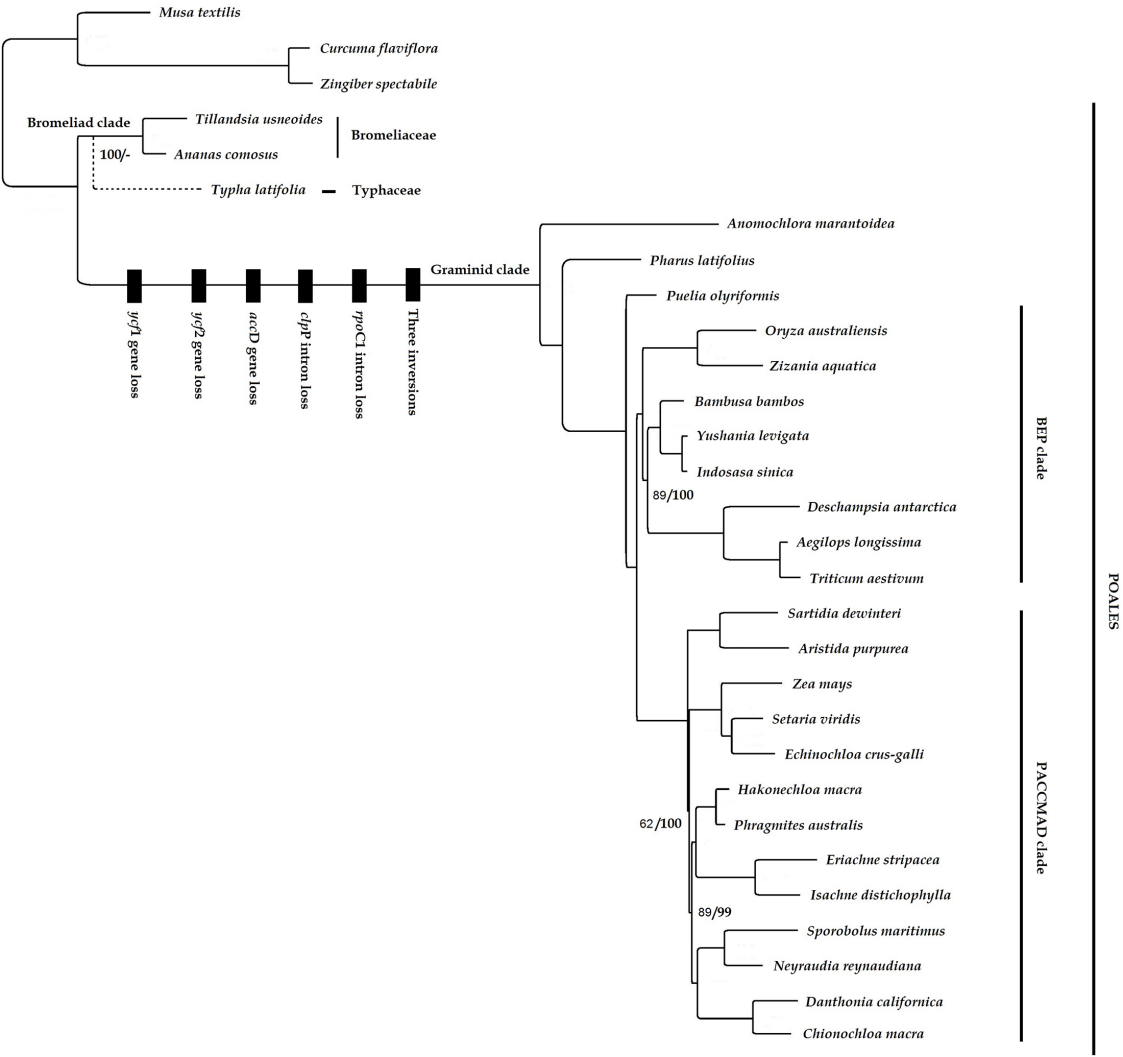
Maximum likelihood (ML) tree of 80 protein coding genes of Poales. Tree of the parsimony analysis was congruent with the ML tree displayed. Topological differences are indicated by dashed lines restricted to the sister grouping of Typhaceae resolved only with parsimony. Bootstrap values less than 100 are shown at nodes for parsimony/ML. Genomic changes are indicated by black bars.

The data matrix assembled for phylogenetic analysis consisted of 30 plastid genomes and 80 genes. All parsimony analyses resulted in one parsimonious tree (S1 Fig) with 31,864 steps and with a CI (consistency index) of 0.64 [68] and RI (retention index) of 0.80 [69]. Maximum likelihood (ML) analysis by RAxML produced a tree with—lnL of -341,682.16. The analysis carried out by removing redundant and lost genes for example *ycf* resulted in similar topology (results not shown). The ML and parsimony trees were congruent with each other and with previous phylogenetic hypotheses (Fig 7).

This included a Graminid clade with a long branch due higher substitution rate accompanied by several genomic changes such as the loss of *clp*P, *rpo*C1 introns and *acc*D, *ycf*1/2 genes and the occurrence of three major inversions [48], and three basal lineages (Anomochlooideae, Pharoideae and Puelioideae), two major clades comprising the BEP clade (Bambusoideae, Ehrhartoideae, and Pooideae) and the PACCMAD clade (Panicoideae, Arundinoideae, Chloridoideae, Centothecoideae, Micrairoideae, Aristidoideae, and Danthonioideae) [70]. Bromeliaceae and Typhaceae, the earliest diverging lineages of Poales were resolved as a polytomy as in previous studies [8, 61, 71] and a united Bromeliad clade [72] was not found in our ML analyses but only in the parsimony analysis. This could be caused by high rate heterogeneity well known for Poales, which causes several long branches relative to other monocots. This, in combination with poor taxon sampling, may result in poorly resolved phylogenies [8]. The two families have a deep split estimated to date to the Late Cretaceous. Pollen grains from Typhaceae are from the Campanian (74–84 Ma), and molecular clock estimates show similar age, 80.9 Ma for the group. The ancestral poalean habitat was most likely dry and sunny and dispersal to wetlands started at the beginning of the Late Cretaceous and have occurred repeatedly. In Bromeliaceae the appearance of shade and wet lineages with CAM photosynthesis occurred only in the Late Miocene 5 Ma [72].

### Selection pressure on the Spanish moss chloroplast genome

To test for signatures of selection we carried out codon-based Z tests by calculating dN/dS values for the selected 80 protein-coding genes across all ten plastid genomes. [73,74]. Values of P less than 0.05 were considered significant at the 5% level and are highlighted in S5 Table. Our findings are generally consistent with the studies of plastid genome evolution of grasses [47, 48]. We found that photosynthetic genes evolve slower than ribosomal protein genes and they appear to be under stronger purifying selection. Synonymous nucleotide substitutions in the plastid genome are generally relatively slow and lineage dependent. Non-synonymous DNA substitutions are even slower due to purifying selection acting on genes as shown in the material studied here. Positive selection is anticipated to speed up non-synonymous substitution rates, whereas synonymous rates are likely to be unaffected.

A recent study found signs of positive selection on *rps*7 gene in pineapple [47]. In the Spanish moss codon-based Z tests also showed significant positive selection for the *rps* gene family in two cases out of ten plastid genomes compared with Spanish moss. A closer inspection of the ribosomal protein small subunit gene group revealed five out of ten genomes compared to the *Tillandsia usneoides* showing p-values less than 0.05 for *rps*7 (S6 Table). Tests were extended over the entire data set of 30 plastid genomes used in the phylogenetic analyses, where 12 species showed significantly higher number of non-synonymous substitution at p-value less than 0.05 for *rps*7. However, when a more stringent significance level is applied (p<0.01) these values become insignificant. As a further step we were interested to find which codons are under positive selection, and to generate robust results confirm codon-based Z tests with other methods. With the default settings and using the constructed best scoring maximum-likelihood tree as input, models of fast unconstrained Bayesian AppRoximation (FUBAR)[39], mixed effects model evolution (MEME)[40], branch-site unrestricted statistical test for episodic diversification (BUSTED)[41] in Hyphy package [37] were also used for conducting the parallel analyses. FUBAR is a codon-based maximum likelihood method that allows *dN*/*dS* (*ω*) to vary over each codon across a gene according to a number of predefined site classes given *a priori*. This allows for testing codons for positive (*dN*/*dS* > 1) or purifying selection (*dN*/*dS* < 1) [39]. While FUBAR posits that e.g. positive selection remains constant throughout time (affects most lineages in a phylogenetic tree) the MEME (Mixed Effects Model of Evolution) test allows the distribution of *ω* to vary over sites and moreover from branch to branch, which makes it possible to detect episodic selection [41]. None of the parallel analysis have found evidence of positive selection at the codon positions. The branch leading to the Bromeliad lineage is short containing only two non-synonymous substitutions total while selection in Poales seems to be episodic and concentrated on the long-branch leading to grasses it is highly unlikely that the *rps*7 gene contains any definitive signal for specific sites under selection as previously reported [47].

We also tested for relaxed selection in a codon-based phylogenetic framework using RELAX [75] on all plastid genes since in a recent study Wicke et al. [76] reported the relaxation of purifying selection of these gene families as an adaptation to parasitic lifestyle. They showed that relaxed purifying selection of plastid genes are directly linked to obligate parasitism and evolutionary rates, while selection pressure coevolves with structural changes of the parasitic lifestyle. The transition to obligate parasitism appears in a stepwise manner, where first functional constraints on genes are relaxed, which is later followed by functional reduction processes and the elevation of evolutionary rates [76]. RELAX first estimates ω among three rate classes for each branch using a branch site-random effects likelihood (BS-REL) method and then fits a parameter *k* indicating the strength of selection [76,77]. ω rate classes are transformed by raising to the power of *k* (ω^*k*^), such that *k* > 1 pushes high and low classes away from 1 (indicative of strong selection) or *k* < 1 scales the high and low rate classes toward 1 (indicative of relaxed selection) [77]. We observed no relaxing selection in plastid genes in the Spanish moss compared to parasitic plants, showing that the epiphytic lifestyle has no selection pressure on plastid evolution.

### Evolution of the *acc*D gene

The *acc*D gene encodes one of four subunits of the acetyl-CoA carboxylase enzyme in most chloroplasts. It is crucial for the regulation of the rate of *de novo* fatty acid biosynthesis in plants. In cases where the *acc*D gene has been completely or partially lost it has been replaced by a nuclear-encoded ACC enzyme [78] with several studies suggesting that it is essential for fatty acid biosynthesis [79]. The *acc*D gene has been maintained even in the non-photosynthetic parasitic plant *Epifagus virginiana* (L.) W. P. C. Barton [80], and in the underground orchid *Rhizanthella gardneri* R. S. Rogers [81]. We observed intact *acc*D genes within the Bromeliad clade as well as in the Commelianales and Zingiberales representatives while we encountered variable forms of this gene in the Graminid clade. Katayama and Ogihara [82] proposed that the *acc*D gene loss occurred at some point before the divergence of Poales and Commelinales. Contrary to this, Konishi et al. [83] showed the presence of *acc*D genes in the Cyperid and Xyrid clades of Poales indicating that the loss occurred at a later point of divergence. Our results based on comparative analyses of the currently available complete chloroplast data support this hypothesis. Bromeliads appear to be basal to the rest of the Poales (Figs 2 and 7) indicating a later point for the *acc*D loss. Harris et al. [59] proposed a point of *acc*D loss past the Eriocaulaceae/Xyridaceae split. The *acc*D gene is reported to be absent in Restionaceae, adding sequence data for the Restiid clade, especially for Anarthriaceae and Centrolepidaceae could refine the status of *acc*D gene in these families.

## Conclusions

Comparison of chloroplast genome organization not only provide us with valuable information for understanding the processes of chloroplast evolution, but also gives insights into the mechanisms underlying genomic rearrangements. We emphasize that additional bromeliad plastid genome sequences are needed to better understand the evolution and structural development of species in this clade. Investigation of plastid genome structure of Poales could trigger further breakthroughs in applied sciences. Understanding of chloroplast biology has potential to shed light on intracellular gene transfer, conservation, diversity, and the genetic basis by which chloroplast transgenes can be engineered to enhance plant agronomic traits or to produce high-value agricultural or biomedical products. The plastid genome is attractive for genetic engineering because it contains genes that are important for pest and disease resistance plus those of importance for herbicide-tolerance traits. One example in agriculture could be herbicide (resistance) research as the family contains many important crops. Cyclohexanediones (CHD) and aryloxyphenoxypropionates (AOPP) have become essential for the control of grass weeds in a variety of dicot crop fields [84]. These herbicides inhibit the acetyl-CoA carboxylase (ACCase) and are selective to monocots. The main reason for this selectivity is the loss of the prokaryotic form of the *acc*D gene in the plastid genome—as also shown in our study. However, selectivity may vary since the loss of the *acc*D is characteristic only for grasses but not to all monocots. We hope that more publicly available Poales plastid genomes will open up new possibilities for development in various fields of applied science. Plastid engineering can also be useful to develop resistance to various abiotic and biotic stress factors based on discovered resilience traits.

## Supporting information

S1 Fig Parsimony phylogenetic tree of 80 protein coding genes of Poales. Bootstrap replicates were obtained from 1,000 replications.
(PDF)

S1 File MAFFT alignment of 80 protein-coding genes of Poales. (FASTA)

S2 File MAFFT alignment of 78 protein-coding genes of Poales. (FASTA)

S3 File MISA annotation file for simple sequence repeats. (MISA)

S1 Table GenBank accession numbers of plastid genomes used in the current study. (XLSX)

S2 Table Primers designed to verify the orientation of inverted repeats. (XLSX)

S3 Table Total number of simple sequence repeats (SSRs) in the Spanish moss genome. (DOCX)

S4 Table Estimate of average evolutionary divergence over 80 protein-coding gene sequences from Poales. (DOCX)

S5 Table Codon-based Z test of selection (neutral, positive, purifying) averaging over all selected sequence pairs. (DOCX)

S6 Table Codon-based Z test of positive selection of selected ribosomal protein small subunit gene 7 averaging over all selected sequence pairs and all plastid genomes included in the analysis. (DOCX)
