# Supplementary figures and images for: The complete chloroplast genome sequence of the CAM epiphyte Spanish moss (Tillandsia usneoides, Bromeliaceae) and its comparative analysis
Source: PLoS One. 2017 Nov 2;12(11):e0187199. doi: 10.1371/journal.pone.0187199 (PMC5667773; doi:10.1371/journal.pone.0187199)

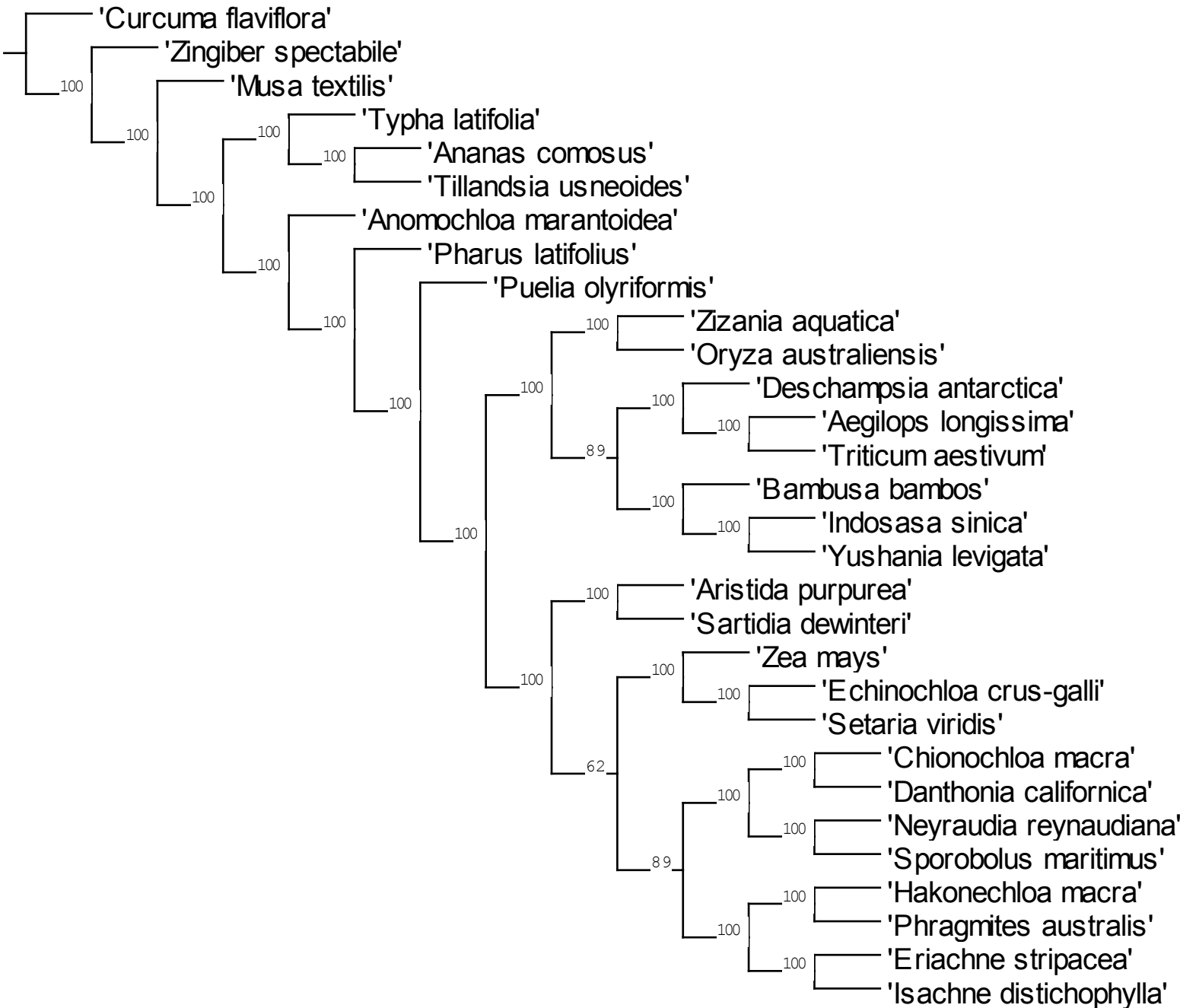

Supplement: S1 Fig — Bootstrap replicates were obtained from 1,000 replications. (PDF) [file pone.0187199.s001.pdf]
