# Supplementary material for: The complete chloroplast genome sequence of the CAM epiphyte Spanish moss (Tillandsia usneoides, Bromeliaceae) and its comparative analysis
Source: PLoS One. 2017 Nov 2;12(11):e0187199. doi: 10.1371/journal.pone.0187199 (PMC5667773; doi:10.1371/journal.pone.0187199)
Supplement: S3 Table — (DOCX) [file pone.0187199.s007.docx]

**Table S3.** Total number of perfect simple sequence repeats (SSRs) identified within the chloroplast genome of *Tillandisa usneoidies*

| **Repeats** | **3** | **4** | **5** | **6** | **7** | **8** | **9** | **10** | **11** | **12** | **13** | **14** | **15** | **16** | **17** | **Total** |
| --- | --- | --- | --- | --- | --- | --- | --- | --- | --- | --- | --- | --- | --- | --- | --- | --- |
| A/T | - | - | - | - | 140 | 43 | 27 | 27 | 11 | 4 | 4 | 7 |  | 1 | 2 | 266 |
| C/G | - | - | - | - | 15 | 5 | 1 |  |  |  |  |  |  |  |  | 21 |
| AC/GT | - | 6 |  |  |  |  |  |  |  |  |  |  |  |  |  | 6 |
| AG/CT | - | 16 | 2 |  |  |  |  |  |  |  |  |  |  |  |  | 18 |
| AT/TA | - | 33 | 5 | 2 | 2 |  |  |  | 1 |  |  |  |  |  |  | 43 |
| AAC/GTT | 11 | 1 |  |  |  |  |  |  |  |  |  |  |  |  |  | 12 |
| AAG/CTT | 19 | 1 |  |  |  |  |  |  |  |  |  |  |  |  |  | 20 |
| AAT/ATT | 12 | 3 |  |  |  |  |  |  |  |  |  |  |  |  |  | 15 |
| ACC/GGT | 2 |  |  |  |  |  |  |  |  |  |  |  |  |  |  | 2 |
| ACT/AGT | 1 |  |  |  |  |  |  |  |  |  |  |  |  |  |  | 1 |
| AGC/CTG | 4 |  |  |  |  |  |  |  |  |  |  |  |  |  |  | 4 |
| AGG/CCT | 1 |  |  |  |  |  |  |  |  |  |  |  |  |  |  | 1 |
| ATC/ATG | 6 |  |  |  |  |  |  |  |  |  |  |  |  |  |  | 6 |
| AAAG/CTTT | 3 |  |  |  |  |  |  |  |  |  |  |  |  |  |  | 3 |
| AAAT/ATTT | 7 | 1 |  |  |  |  |  |  |  |  |  |  |  |  |  | 8 |
| AACT/AGTT | 1 |  |  |  |  |  |  |  |  |  |  |  |  |  |  | 1 |
| AATC/ATTG | 1 |  |  |  |  |  |  |  |  |  |  |  |  |  |  | 1 |
| AATG/ATTC | 2 |  |  |  |  |  |  |  |  |  |  |  |  |  |  | 2 |
| AAAAT/ATTTT | 1 |  |  |  |  |  |  |  |  |  |  |  |  |  |  | 1 |
| AAAAGT/ACTTTT | 1 |  |  |  |  |  |  |  |  |  |  |  |  |  |  | 1 |
|  |  |  |  |  |  |  |  |  |  |  |  |  |  |  |  | 432 |
