# Supplementary material for: The complete chloroplast genome sequence of the CAM epiphyte Spanish moss (Tillandsia usneoides, Bromeliaceae) and its comparative analysis
Source: PLoS One. 2017 Nov 2;12(11):e0187199. doi: 10.1371/journal.pone.0187199 (PMC5667773; doi:10.1371/journal.pone.0187199)
Supplement: S4 Table — (DOCX) [file pone.0187199.s008.docx]

**Table S4** - Estimates of average evolutionary divergence over 80 protein coding-gene sequences from Poales. Standard error estimate(s) are shown in the fourth column and were obtained by a bootstrap procedure (1000 replicates). Analyses were conducted using the Kimura 2-parameter model. Codon positions included were 1st + 2nd + 3rd + Noncoding. All positions with less than 95 % site coverage were eliminated. That is, fewer than 5 % alignment gaps, missing data, and ambiguous bases were allowed at any position.

| **Functional Group** | **Gene** | **Location** | **d** | **S.E.** | **Aligned Length** | **Length** | **Missing** |
| --- | --- | --- | --- | --- | --- | --- | --- |
| Ribosomal protein small subunit | rps2 | LSC | 0.064 | 0.005 | 721 | 711-720 |  |
|  | rps3 | LSC | 0.092 | 0.007 | 735 | 657-720 |  |
|  | rps4 | LSC | 0.071 | 0.006 | 606 | 600-606 |  |
|  | rps7 | IR | 0.028 | 0.004 | 471 | 468-471 |  |
|  | rps8 | LSC | 0.070 | 0.007 | 412 | 400-412 |  |
|  | rps11 | LSC | 0.082 | 0.008 | 441 | 417-432 |  |
|  | rps12 | IR | 0.018 | 0.002 | 916 | 912-915 |  |
|  | rps14 | LSC | 0.057 | 0.007 | 315 | 303-312 |  |
|  | rps15 | SSC | 0.078 | 0.009 | 323 | 273-316 |  |
|  | rps16 | LSC | 0.078 | 0.005 | 1420 | 1013-1161 |  |
|  | **rps18** | LSC | **0.105** | 0.011 | 567 | 346-517 |  |
|  | rps19 | IR | 0.071 | 0.010 | 634 | 168-589 |  |
| Ribosomal protein large subunit | rpl2 | IR | 0.024 | 0.003 | 1505 | 498-1491 |  |
|  | rpl14 | LSC | 0.061 | 0.008 | 372 | 302-372 |  |
|  | rpl16 | LSC | 0.077 | 0.008 | 1811 | 402-1514 | *Hakonochlea macra* |
|  | rpl20 | LSC | 0.077 | 0.009 | 450 | 354-429 |  |
|  | **rpl22** | LSC | **0.101** | 0.010 | 477 | 330-474 |  |
|  | rpl23 | IR | 0.041 | 0.007 | 300 | 282-297 |  |
|  | **rpl32** | IR | **0.119** | 0.015 | 208 | 174-201 |  |
|  | rpl33 | LSC | 0.097 | 0.013 | 201 | 201 | *Pharus latifolius* |
|  | rpl36 | LSC | 0.070 | 0.015 | 114 | 114 |  |
| Subunits of RNA polymerase | rpoA | LSC | 0.077 | 0.004 | 1059 | 1014-1038 |  |
|  | rpoB | LSC | 0.062 | 0.003 | 3297 | 3219-3237 |  |
|  | rpoC1 | LSC | 0.076 | 0.004 | 2858 | 2031-2794 |  |
|  | rpoC2 | LSC | 0.083 | 0.002 | 4900 | 4095-4614 |  |
| Photosystem I | psaA | LSC | 0.044 | 0.002 | 2256 | 2253-2256 |  |
|  | psaB | LSC | 0.039 | 0.002 | 2208 | 2205-2208 |  |
|  | psaC | SSC | 0.057 | 0.009 | 246 | 246 |  |
|  | psaI | LSC | 0.062 | 0.013 | 111 | 105-111 |  |
|  | psaJ | LSC | 0.056 | 0.012 | 135 | 129-135 |  |
| Photosystem II | psbA | LSC | 0.038 | 0.003 | 1062 | 1062 |  |
|  | psbB | LSC | 0.045 | 0.003 | 1527 | 1527 |  |
|  | psbC | LSC | 0.047 | 0.003 | 1422 | 1422 |  |
|  | psbD | LSC | 0.044 | 0.003 | 1062 | 1062 |  |
|  | psbE | LSC | 0.028 | 0.005 | 252 | 249 |  |
|  | psbF | LSC | 0.044 | 0.011 | 123 | 120-123 |  |
|  | psbH | LSC | 0.083 | 0.011 | 228 | 222-228 |  |
|  | psbI | LSC | 0.043 | 0.010 | 111 | 111 |  |
|  | psbJ | LSC | 0.044 | 0.010 | 123 | 123 |  |
|  | psbK | LSC | 0.065 | 0.010 | 192 | 186-192 |  |
|  | psbL | LSC | 0.014 | 0.005 | 117 | 116-117 |  |
|  | psbM | LSC | 0.051 | 0.012 | 105 | 105 |  |
|  | psbN | LSC | 0.029 | 0.008 | 132 | 132 |  |
|  | psbT | LSC | 0.048 | 0.014 | 117 | 101-117 |  |
|  | psbZ | LSC | 0.047 | 0.009 | 194 | 159-194 |  |
| Cytochrome b/f complex | petA | LSC | 0.056 | 0.004 | 963 | 963 |  |
|  | petB | LSC | 0.041 | 0.004 | 1658 | 648-1530 |  |
|  | petD | LSC | 0.073 | 0.004 | 1378 | 1206-1263 |  |
|  | petG | LSC | 0.040 | 0.011 | 117 | 114-115 |  |
|  | petL | LSC | 0.036 | 0.010 | 96 | 96 |  |
|  | petN | LSC | 0.033 | 0.010 | 90 | 90 |  |
| ATP synthase | atpA | LSC | 0.060 | 0.003 | 1524 | 1515-1524 |  |
|  | atpB | LSC | 0.061 | 0.003 | 1497 | 1488-1497 |  |
|  | atpE | LSC | 0.075 | 0.007 | 414 | 405-414 |  |
|  | atpF | LSC | 0.076 | 0.004 | 1596 | 1344-1406 |  |
|  | atpH | LSC | 0.034 | 0.007 | 246 | 246 |  |
|  | atpI | LSC | 0.047 | 0.005 | 744 | 744 |  |
| NADH-dehydrogenase | ndhA | SSC | 0.082 | 0.005 | 2434 | 1089-2210 |  |
|  | ndhB | IR | 0.014 | 0.001 | 2253 | 2233-2249 |  |
|  | ndhC | LSC | 0.056 | 0.007 | 363 | 363 |  |
|  | ndhD | SSC | 0.075 | 0.004 | 1506 | 1503-1506 |  |
|  | ndhE | SSC | 0.062 | 0.008 | 307 | 291-306 |  |
|  | **ndhF** | SSC | **0.104** | 0.004 | 2268 | 2202-2229 |  |
|  | ndhG | SSC | 0.085 | 0.007 | 531 | 531 |  |
|  | ndhH | SSC | 0.073 | 0.005 | 1194 | 1182-1188 |  |
|  | ndhI | SSC | 0.069 | 0.006 | 549 | 537-549 |  |
|  | ndhJ | LSC | 0.057 | 0.005 | 480 | 480 |  |
|  | ndhK | LSC | 0.059 | 0.005 | 915 | 669-879 |  |
| Large subunit Rubisco | rbcL | LSC | 0.065 | 0.004 | 1470 | 1428-1464 |  |
| Translation initiation factor IF-1 | **infA** | LSC | **0.115** | 0.016 | 357 | 234-351 |  |
| Acetyl-CoA carboxylase | **accD** | LSC | **0.297** | 0.065 | 1828 | 250-1639 |  |
| Cytochrome c biogenesis | ccsA | SSC | 0.082 | 0.005 | 1040 | 927-990 |  |
| Maturase | **matK** | LSC | **0.119** | 0.005 | 1684 | 1536-1632 |  |
| ATP-dependent protease | **clpP** | LSC | **0.117** | 0.008 | 2302 | 612-2160 |  |
| Inner membrane protein | cemA | LSC | 0.095 | 0.006 | 705 | 690-699 |  |
| Conserved hypothetical chloroplast ORF | **ycf1** | SSC | **0.130** | 0.009 | 6243 | 653-5712 |  |
|  | **ycf2** | IR | **0.204** | 0.068 | 7236 | 130-6919 |  |
|  | ycf3 | LSC | 0.069 | 0.003 | 2359 | 1934-2062 |  |
|  | ycf4 | LSC | 0.060 | 0.005 | 569 | 552-569 |  |
|  | ycf15 | IR | 0.040 | 0.010 | 634 | 168-589 |  |
