# Supplementary material for: The complete chloroplast genome sequence of the CAM epiphyte Spanish moss (Tillandsia usneoides, Bromeliaceae) and its comparative analysis
Source: PLoS One. 2017 Nov 2;12(11):e0187199. doi: 10.1371/journal.pone.0187199 (PMC5667773; doi:10.1371/journal.pone.0187199)
Supplement: S5 Table — (DOCX) [file pone.0187199.s009.docx]

**Table S5** – Codon-based Z test of selection (neutral, positive, purifying) averaging over all selected sequence pairs. The variance of the difference was computed using the bootstrap method (1000 replicates). Analyses were conducted using the Nei-Gojobori method. Gene groups were categorized according to gene function or subunits that form a functional complex; values were, respectively, combined for *atp*-, *ndh*-, *pet-*, *psa*-, *psb*-,*rpl*-, *rps*-, and *rpo*-genes.

| **Functional Group** | ***Ananas comosus*** | |  |  |  |  |
| --- | --- | --- | --- | --- | --- | --- |
|  | Neutral (dN = dS) | | Positive (dN > dS) | | Purifying (dN < dS) | |
|  | dN/dS | P-value | dN/dS | P-value | dN/dS | P-value |
| Ribosomal protein small subunit (*rps*) | − 1.683 | 0.005 | − 1.702 | 1 | 1.755 | 0.041 |
| Ribosomal protein large subunit (*rpl*) | 0.702 | 0.484 | 0.684 | 0.248 | − 0.702 | 1 |
| Subunits of RNA polymerase (rpo) | − 4.910 | 0 | − 4.830 | 1 | 5.144 | 0 |
| Photosystem I (*psa*) | − 4.893 | 0 | − 4.800 | 1 | 4.728 | 0 |
| Photosystem II (*psb*) | − 5.304 | 0 | − 5.223 | 1 | 5.146 | 0 |
| Cytochrome b/f complex (*pet*) | − 2.211 | 0.029 | − 2.179 | 1 | 2.208 | 0.015 |
| ATP synthase (*atp*) | − 4.000 | 0 | − 4.152 | 1 | 4.020 | 0 |
| NADH-dehydrogenase (*ndh*) | − 6.077 | 0 | − 6.132 | 1 | 6.167 | 0 |
| Large subunit Rubisco (*rbc*L) | − 2.030 | 0.045 | − 2.038 | 1 | 2.022 | 0.023 |
| Translation initiation factor IF-1 (*inf*A) | − 1.062 | 0.291 | − 1.083 | 1 | 1.039 | 0.150 |
| Acetyl-CoA carboxylase (*acc*D) | − 0.677 | 0.500 | 1 | − 0.687 | 0.659 | 0.256 |
| Cytochrome c biogenesis (*ccs*A) | − 2.456 | 0.015 | − 2.402 | 1 | 2.512 | 0.007 |
| Maturase (*mat*K) | − 2.010 | 0.047 | − 2 | 1 | 1.989 | 0.024 |
| ATP-dependent protease (*clp*P) | − 0.274 | 0.785 | − 0.258 | 1 | 0.268 | 0.395 |
| Inner membrane protein (*cem*A) | − 2.083 | 0.039 | − 1.997 | 1 | 2.103 | 0.019 |
| Conserved hypothetical chloroplast ORF (*ycf*) | − 3.057 | 0.003 | − 3.098 | 1 | 3.077 | 0.001 |

| **Functional Group** | ***Bambusa bambos*** | |  |  |  |  |
| --- | --- | --- | --- | --- | --- | --- |
|  | Neutral (dN = dS) | | Positive (dN > dS) | | Purifying (dN < dS) | |
|  | dN/dS | P-value | dN/dS | P-value | dN/dS | P-value |
| Ribosomal protein small subunit (*rps*) | − 3.277 | 0.001 | − 3.158 | 1 | 3.318 | 0.001 |
| Ribosomal protein large subunit (*rpl*) | − 1.716 | 0.089 | − 1.700 | 1 | 1.659 | 0.050 |
| Subunits of RNA polymerase (rpo) | − 6.010 | 0 | − 6.064 | 1 | 5.887 | 0 |
| Photosystem I (*psa*) | − 15.707 | 0 | − 15.700 | 1 | 16.091 | 0 |
| Photosystem II (*psb*) | − 17.563 | 0 | − 17.257 | 1 | 17.382 | 0 |
| Cytochrome b/f complex (*pet*) | − 7.637 | 0 | − 7.485 | 1 | 7.576 | 0 |
| ATP synthase (*atp*) | − 4.810 | 0 | − 4.884 | 1 | 4.533 | 0 |
| NADH-dehydrogenase (*ndh*) | 0.091 | 0.928 | 0.093 | 0.463 | − 0.094 | 1 |
| Large subunit Rubisco (*rbc*L) | − 10.646 | 0 | − 10.570 | 1 | 10.243 | 0 |
| Translation initiation factor IF-1 (*inf*A) | − 4.530 | 0 | − 4.253 | 1 | 4.554 | 0 |
| Acetyl-CoA carboxylase (*acc*D) | − 1.116 | 0.267 | − 1.105 | 1 | 1.082 | 0.141 |
| Cytochrome c biogenesis (*ccs*A) | − 4.922 | 0 | − 4.838 | 1 | 4.974 | 0 |
| Maturase (*mat*K) | − 6.102 | 0 | − 6.221 | 1 | 6.216 | 0 |
| ATP-dependent protease (*clp*P) | − 2.555 | 0.012 | − 2.428 | 1 | 2.449 | 0.008 |
| Inner membrane protein (*cem*A) | − 1.605 | 0.111 | − 1.552 | 1 | 1.578 | 0.059 |
| Conserved hypothetical chloroplast ORF (*ycf*) | − 1.491 | 0.139 | − 1.470 | 1 | 1.508 | 0.067 |

| **Functional Group** | ***Deschampsia antarctica*** | | |  |  |  |
| --- | --- | --- | --- | --- | --- | --- |
|  | Neutral (dN = dS) | | Positive (dN > dS) | | Purifying (dN < dS) | |
|  | dN/dS | P-value | dN/dS | P-value | dN/dS | P-value |
| Ribosomal protein small subunit (*rps*) | − 6.873 | 0 | − 7.172 | 1 | 6.824 | 0 |
| Ribosomal protein large subunit (*rpl*) | − 3.274 | 0.001 | − 3.308 | 1 | 3.271 | 0.001 |
| Subunits of RNA polymerase (rpo) | − 6.656 | 0 | − 6.612 | 1 | 6.600 | 0 |
| Photosystem I (*psa*) | − 18.195 | 0 | − 17.892 | 1 | 18.367 | 0 |
| Photosystem II (*psb*) | − 18.029 | 0 | 18.968 | 1 | 18.504 | 0 |
| Cytochrome b/f complex (*pet*) | − 4.833 | 0 | − 4.821 | 1 | 5.505 | 0 |
| ATP synthase (*atp*) | 0.529 | 0.598 | 0.510 | 0.306 | − 0.498 | 1 |
| NADH-dehydrogenase (*ndh*) | 5.778 | 0 | 6.035 | 0 | − 5.727 | 1 |
| Large subunit Rubisco (*rbc*L) | − 10.722 | 0 | − 10.602 | 1 | 10.986 | 0 |
| Translation initiation factor IF-1 (*inf*A) | − 4.595 | 0 | − 4.588 | 1 | 4.406 | 0 |
| Acetyl-CoA carboxylase (*acc*D) | − 1.077 | 0.284 | − 1.125 | 1 | 1.144 | 0.127 |
| Cytochrome c biogenesis (*ccs*A) | − 5.505 | 0 | − 5.705 | 1 | 5.589 | 0 |
| Maturase (*mat*K) | − 6.330 | 0 | − 6.411 | 1 | 6.193 | 0 |
| ATP-dependent protease (*clp*P) | − 3.113 | 0.002 | − 3.008 | 1 | 3.166 | 0.001 |
| Inner membrane protein (*cem*A) | − 1.721 | 0.088 | − 1.645 | 1 | 1.729 | 0.043 |
| Conserved hypothetical chloroplast ORF (*ycf*) | − 1.902 | 0.060 | − 1.915 | 1 | 1.831 | 0.035 |

| **Functional Group** | ***Musa textilis*** | |  |  |  |  |
| --- | --- | --- | --- | --- | --- | --- |
|  | Neutral (dN = dS) | | Positive (dN > dS) | | Purifying (dN < dS) | |
|  | dN/dS | P-value | dN/dS | P-value | dN/dS | P-value |
| Ribosomal protein small subunit (*rps*) | − 1.316 | 0.191 | − 1.336 | 1 | 1.321 | 0.094 |
| Ribosomal protein large subunit (*rpl*) | − 1.105 | 0.272 | − 1.120 | 1 | 1.061 | 0.145 |
| Subunits of RNA polymerase (rpo) | − 7.097 | 0 | − 7.373 | 1 | 6.897 | 0 |
| Photosystem I (*psa*) | − 12.934 | 0 | − 12.934 | 1 | 13.130 | 0 |
| Photosystem II (*psb*) | − 13.147 | 0 | − 13.093 | 1 | 13.296 | 0 |
| Cytochrome b/f complex (*pet*) | − 6.346 | 0 | − 6.241 | 1 | 6.090 | 0 |
| ATP synthase (*atp*) | − 4.507 | 0 | − 4.525 | 1 | 4.598 | 0 |
| NADH-dehydrogenase (*ndh*) | 0.070 | 0.944 | 0.070 | 0.472 | − 0.070 | 1 |
| Large subunit Rubisco (*rbc*L) | − 6.704 | 0 | − 6.679 | 1 | 6.543 | 0 |
| Translation initiation factor IF-1 (*inf*A) | − 3.533 | 0.001 | − 3.453 | 1 | 3.678 | 0 |
| Acetyl-CoA carboxylase (*acc*D) | − 0.360 | 0.719 | − 0.365 | 1 | 0.363 | 0.359 |
| Cytochrome c biogenesis (*ccs*A) | − 3.920 | 0 | − 3.776 | 1 | 3.712 | 0 |
| Maturase (*mat*K) | − 5.120 | 0 | − 5.241 | 1 | 5.241 | 0 |
| ATP-dependent protease (*clp*P) | 0.022 | 0.983 | 0.023 | 0.491 | − 0.022 | 1 |
| Inner membrane protein (*cem*A) | − 4.537 | 0 | − 4.334 | 1 | 4.298 | 0 |
| Conserved hypothetical chloroplast ORF (*ycf*) | − 1.520 | 0.131 | − 1.539 | 1 | 1.506 | 0.067 |

| **Functional Group** | ***Triticum aestivum*** | |  |  |  |  |
| --- | --- | --- | --- | --- | --- | --- |
|  | Neutral (dN = dS) | | Positive (dN > dS) | | Purifying (dN < dS) | |
|  | dN/dS | P-value | dN/dS | P-value | dN/dS | P-value |
| Ribosomal protein small subunit (*rps*) | 2.029 | 0.045 | 2.027 | 0.022 | − 2.027 | 1 |
| Ribosomal protein large subunit (*rpl*) | 0.001 | 0.999 | 0.001 | 0.499 | − 0.001 | 1 |
| Subunits of RNA polymerase (rpo) | − 6.694 | 0 | − 6.568 | 1 | 6.617 | 0 |
| Photosystem I (*psa*) | − 18.070 | 0 | − 17.528 | 1 | 17.605 | 0 |
| Photosystem II (*psb*) | − 18.168 | 0 | − 18.201 | 1 | 17.939 | 0 |
| Cytochrome b/f complex (*pet*) | − 8.658 | 0 | − 8.431 | 1 | 8.544 | 0 |
| ATP synthase (*atp*) | 0.263 | 0.793 | 0.269 | 0.394 | − 0.273 | 1 |
| NADH-dehydrogenase (*ndh*) | − 18.972 | 0 | − 18.985 | 1 | 18.884 | 0 |
| Large subunit Rubisco (*rbc*L) | − 11.294 | 0 | − 11.503 | 1 | 11.034 | 0 |
| Translation initiation factor IF-1 (*inf*A) | − 4.978 | 0 | − 4.780 | 1 | 4.937 | 0 |
| Acetyl-CoA carboxylase (*acc*D) | − 0.734 | 0.464 | − 0.753 | 1 | 0.791 | 0.215 |
| Cytochrome c biogenesis (*ccs*A) | − 4.948 | 0 | − 4.818 | 1 | 4.967 | 0 |
| Maturase (*mat*K) | − 7.027 | 0 | − 6.945 | 1 | 7.145 | 0 |
| ATP-dependent protease (*clp*P) | − 2.460 | 0.015 | − 2.444 | 1 | 2.477 | 0.007 |
| Inner membrane protein (*cem*A) | − 1.329 | 0.186 | − 1.406 | 1 | 1.447 | 0.075 |
| Conserved hypothetical chloroplast ORF (*ycf*) | − 0.257 | 0.798 | − 0.249 | 1 | 0.256 | 0.399 |

| **Functional Group** | ***Typha latifolia*** | |  |  |  |  |
| --- | --- | --- | --- | --- | --- | --- |
|  | Neutral (dN = dS) | | Positive (dN > dS) | | Purifying (dN < dS) | |
|  | dN/dS | P-value | dN/dS | P-value | dN/dS | P-value |
| Ribosomal protein small subunit (*rps*) | 1.983 | 0.050 | 1.982 | 0.025 | − 1.942 | 1 |
| Ribosomal protein large subunit (*rpl*) | − 0.574 | 0.567 | − 0.566 | 1 | 0.577 | 0.283 |
| Subunits of RNA polymerase (rpo) | − 6.426 | 0 | − 6.250 | 1 | 6.501 | 0 |
| Photosystem I (*psa*) | − 10.891 | 0 | − 11.363 | 1 | 11.237 | 0 |
| Photosystem II (*psb*) | − 12.359 | 0 | − 12.239 | 1 | 12.374 | 0 |
| Cytochrome b/f complex (*pet*) | − 4.156 | 0 | − 4.186 | 1 | 4.182 | 0 |
| ATP synthase (*atp*) | − 7.937 | 0 | − 7.722 | 1 | 7.445 | 0 |
| NADH-dehydrogenase (*ndh*) | − 12.612 | 0 | − 12.757 | 1 | 12.335 | 0 |
| Large subunit Rubisco (*rbc*L) | − 5.969 | 0 | − 5.865 | 1 | 5.839 | 0 |
| Translation initiation factor IF-1 (*inf*A) | − 2.491 | 0.014 | − 2.615 | 1 | 2.629 | 0.005 |
| Acetyl-CoA carboxylase (*acc*D) | − 0.873 | 0.384 | − 0.861 | 1 | 0.870 | 0.193 |
| Cytochrome c biogenesis (*ccs*A) | − 4.218 | 0 | − 4.153 | 1 | 4.311 | 0 |
| Maturase (*mat*K) | − 3.751 | 0 | − 3.677 | 1 | 3.927 | 0 |
| ATP-dependent protease (*clp*P) | − 3.047 | 0.003 | − 2.970 | 1 | 3.018 | 0.002 |
| Inner membrane protein (*cem*A) | − 3.902 | 0 | − 4.032 | 1 | 4.015 | 0 |
| Conserved hypothetical chloroplast ORF (*ycf*) | − 5.169 | 0 | − 5.088 | 1 | 5.163 | 0 |

| **Functional Group** | ***Typha latifolia*** | |  |  |  |  |
| --- | --- | --- | --- | --- | --- | --- |
|  | Neutral (dN = dS) | | Positive (dN > dS) | | Purifying (dN < dS) | |
|  | dN/dS | P-value | dN/dS | P-value | dN/dS | P-value |
| Ribosomal protein small subunit (*rps*) | 1.983 | 0.050 | 1.982 | 0.025 | − 1.942 | 1 |
| Ribosomal protein large subunit (*rpl*) | − 0.574 | 0.567 | − 0.566 | 1 | 0.577 | 0.283 |
| Subunits of RNA polymerase (rpo) | − 6.426 | 0 | − 6.250 | 1 | 6.501 | 0 |
| Photosystem I (*psa*) | − 10.891 | 0 | − 11.363 | 1 | 11.237 | 0 |
| Photosystem II (*psb*) | − 12.359 | 0 | − 12.239 | 1 | 12.374 | 0 |
| Cytochrome b/f complex (*pet*) | − 4.156 | 0 | − 4.186 | 1 | 4.182 | 0 |
| ATP synthase (*atp*) | − 7.937 | 0 | − 7.722 | 1 | 7.445 | 0 |
| NADH-dehydrogenase (*ndh*) | − 12.612 | 0 | − 12.757 | 1 | 12.335 | 0 |
| Large subunit Rubisco (*rbc*L) | − 5.969 | 0 | − 5.865 | 1 | 5.839 | 0 |
| Translation initiation factor IF-1 (*inf*A) | − 2.491 | 0.014 | − 2.615 | 1 | 2.629 | 0.005 |
| Acetyl-CoA carboxylase (*acc*D) | − 0.873 | 0.384 | − 0.861 | 1 | 0.870 | 0.193 |
| Cytochrome c biogenesis (*ccs*A) | − 4.218 | 0 | − 4.153 | 1 | 4.311 | 0 |
| Maturase (*mat*K) | − 3.751 | 0 | − 3.677 | 1 | 3.927 | 0 |
| ATP-dependent protease (*clp*P) | − 3.047 | 0.003 | − 2.970 | 1 | 3.018 | 0.002 |
| Inner membrane protein (*cem*A) | − 3.902 | 0 | − 4.032 | 1 | 4.015 | 0 |
| Conserved hypothetical chloroplast ORF (*ycf*) | − 5.169 | 0 | − 5.088 | 1 | 5.163 | 0 |

| **Functional Group** | ***Zea mays*** | |  |  |  |  |
| --- | --- | --- | --- | --- | --- | --- |
|  | Neutral (dN = dS) | | Positive (dN > dS) | | Purifying (dN < dS) | |
|  | dN/dS | P-value | dN/dS | P-value | dN/dS | P-value |
| Ribosomal protein small subunit (*rps*) | − 5.558 | 0 | − 5.730 | 1 | 5.763 | 0 |
| Ribosomal protein large subunit (*rpl*) | − 2.274 | 0.025 | − 2.197 | 1 | 2.236 | 0.014 |
| Subunits of RNA polymerase (rpo) | − 6.781 | 0 | − 6.859 | 1 | 6.859 | 0 |
| Photosystem I (*psa*) | − 17.520 | 0 | − 17.166 | 1 | 16.872 | 0 |
| Photosystem II (*psb*) | − 17.834 | 0 | − 18.544 | 1 | 17.468 | 0 |
| Cytochrome b/f complex (*pet*) | − 5.041 | 0 | − 5.214 | 1 | 5.042 | 0 |
| ATP synthase (*atp*) | − 6.348 | 0 | − 6.563 | 1 | 6.446 | 0 |
| NADH-dehydrogenase (*ndh*) | 0.919 | 0.360 | 0.910 | 0.182 | − 0.935 | 1 |
| Large subunit Rubisco (*rbc*L) | − 11.464 | 0 | − 10.991 | 1 | 10.977 | 0 |
| Translation initiation factor IF-1 (*inf*A) | − 5.629 | 0 | − 5.080 | 1 | 5.168 | 0 |
| Acetyl-CoA carboxylase (*acc*D) | − 0.256 | 0.798 | − 0.264 | 1 | 0.253 | 0.400 |
| Cytochrome c biogenesis (*ccs*A) | − 5.672 | 0 | − 5.379 | 1 | 5.321 | 0 |
| Maturase (*mat*K) | − 6.348 | 0 | − 6.250 | 1 | 6.273 | 0 |
| ATP-dependent protease (*clp*P) | − 2.674 | 0.009 | − 2.662 | 1 | 2.662 | 0.004 |
| Inner membrane protein (*cem*A) | − 2.769 | 0.007 | − 2.975 | 1 | 2.830 | 0.003 |
| Conserved hypothetical chloroplast ORF (*ycf*) | − 1.005 | 0.317 | − 0.924 | 1 | 0.933 | 0.176 |

| **Functional Group** | ***Zingiber spectabile*** | |  |  |  |  |
| --- | --- | --- | --- | --- | --- | --- |
|  | Neutral (dN = dS) | | Positive (dN > dS) | | Purifying (dN < dS) | |
|  | dN/dS | P-value | dN/dS | P-value | dN/dS | P-value |
| Ribosomal protein small subunit (*rps*) | − 1.816 | 0.072 | − 1.762 | 1 | 1.784 | 0.038 |
| Ribosomal protein large subunit (*rpl*) | − 4.707 | 0 | − 4.821 | 1 | 4.617 | 0 |
| Subunits of RNA polymerase (rpo) | − 7.733 | 0 | − 7.894 | 1 | 7.420 | 0 |
| Photosystem I (*psa*) | − 13.904 | 0 | − 14.174 | 1 | 14.958 | 0 |
| Photosystem II (*psb*) | − 15.782 | 0 | − 15.349 | 1 | 16.207 | 0 |
| Cytochrome b/f complex (*pet*) | − 7.433 | 0 | − 7.678 | 1 | 7.381 | 0 |
| ATP synthase (*atp*) | − 8.431 | 0 | − 8.547 | 1 | 8.237 | 0 |
| NADH-dehydrogenase (*ndh*) | 2.098 | 0.038 | 2.042 | 0.022 | − 2.046 | 1 |
| Large subunit Rubisco (*rbc*L) | − 7.913 | 0 | − 7.316 | 1 | 7.659 | 0 |
| Translation initiation factor IF-1 (*inf*A) | − 2.528 | 0.013 | − 2.617 | 1 | 2.588 | 0.005 |
| Acetyl-CoA carboxylase (*acc*D) | − 0.322 | 0.748 | − 0.342 | 1 | 0.330 | 0.371 |
| Cytochrome c biogenesis (*ccs*A) | − 4.312 | 0 | − 4.533 | 1 | 4.309 | 0 |
| Maturase (*mat*K) | − 5.757 | 0 | − 5.664 | 1 | 5.814 | 0 |
| ATP-dependent protease (*clp*P) | − 1.053 | 0.294 | − 1.112 | 1 | 1.069 | 0.144 |
| Inner membrane protein (*cem*A) | − 5.344 | 0 | − 5.007 | 1 | 5.101 | 0 |
| Conserved hypothetical chloroplast ORF (*ycf*) | − 3.834 | 0 | − 3.829 | 1 | 3.857 | 0 |

| **Functional Group** | ***Zizania aquatica*** | |  |  |  |  |
| --- | --- | --- | --- | --- | --- | --- |
|  | Neutral (dN = dS) | | Positive (dN > dS) | | Purifying (dN < dS) | |
|  | dN/dS | P-value | dN/dS | P-value | dN/dS | P-value |
| Ribosomal protein small subunit (*rps*) | − 2.874 | 0.005 | − 2.836 | 1 | 2.882 | 0.002 |
| Ribosomal protein large subunit (*rpl*) | − 0.643 | 0.521 | − 0.630 | 1 | 0.646 | 0.260 |
| Subunits of RNA polymerase (rpo) | − 5.794 | 0 | − 5.776 | 1 | 5.730 | 0 |
| Photosystem I (*psa*) | − 18.119 | 0 | − 17.939 | 1 | 17.796 | 0 |
| Photosystem II (*psb*) | − 19.769 | 0 | − 20.012 | 1 | 19.728 | 0 |
| Cytochrome b/f complex (*pet*) | − 6.354 | 0 | − 6.255 | 1 | 6.600 | 0 |
| ATP synthase (*atp*) | − 3.747 | 0 | − 3.831 | 1 | 3.567 | 0 |
| NADH-dehydrogenase (*ndh*) | − 19.461 | 0 | − 20.172 | 1 | 19.986 | 0 |
| Large subunit Rubisco (*rbc*L) | − 11.427 | 0 | − 11.282 | 1 | 10.890 | 0 |
| Translation initiation factor IF-1 (*inf*A) | − 4.592 | 0 | − 4.542 | 1 | 4.456 | 0 |
| Acetyl-CoA carboxylase (*acc*D) | − 2.193 | 0.030 | − 2.031 | 1 | 2.086 | 0.020 |
| Cytochrome c biogenesis (*ccs*A) | − 6.126 | 0 | − 5.908 | 1 | 5.720 | 0 |
| Maturase (*mat*K) | − 6.886 | 0 | − 6.630 | 1 | 6.620 | 0 |
| ATP-dependent protease (*clp*P) | − 2.342 | 0.021 | − 2.227 | 1 | 2.281 | 0.012 |
| Inner membrane protein (*cem*A) | − 2.475 | 0.015 | − 2.223 | 1 | 2.207 | 0.015 |
| Conserved hypothetical chloroplast ORF (*ycf*) | − 3.324 | 0.001 | − 3.268 | 1 | 3.257 | 0.001 |
