# Supplementary material for: The complete chloroplast genome sequence of the CAM epiphyte Spanish moss (Tillandsia usneoides, Bromeliaceae) and its comparative analysis
Source: PLoS One. 2017 Nov 2;12(11):e0187199. doi: 10.1371/journal.pone.0187199 (PMC5667773; doi:10.1371/journal.pone.0187199)
Supplement: S6 Table — (DOCX) [file pone.0187199.s010.docx]

**Table S6** - Codon-based Z test of positive selection of selected ribosomal protein small subunit gene 7 averaging over all selected sequence pairs and all plastid genomes included in the analyses. The variance of the difference was computed using the bootstrap method (1000 replicates). Analyses were conducted using the Nei-Gojobori method.

|  | *rps*7 | |
| --- | --- | --- |
| Species | Positive (dN > dS) | |
|  | dN/dS | P-value |
| *Aegilops longissima* | 1.742 | 0.042 |
| *Anomochloa marantoidea* | 1.910 | 0.029 |
| *Aristida purpurea* | 1.372 | 0.086 |
| *Chionochloa macra* | 1.840 | 0.034 |
| *Curcuma flavilfora* | − 0.612 | 1 |
| *Danthonia californica* | 1.882 | 0.031 |
| *Echinochloa crus-galli* | 1.296 | 0.099 |
| *Eriachne stripacea* | 1.288 | 0.100 |
| *Hakonechloa macra* | 0.843 | 0.200 |
| *Indosasa sinica* | 1.801 | 0.037 |
| *Isachne distichophylla* | 1.838 | 0.034 |
| *Neyraudia reynaudiana* | 1.834 | 0.035 |
| *Oryza australiensis* | 1.918 | 0.029 |
| *Pharus latifolius* | 1.901 | 0.030 |
| *Phragmites australis* | 1.265 | 0.104 |
| *Puelia olyriformis* | 1.867 | 0.032 |
| *Sartidia dewinteri* | 1.852 | 0.033 |
| *Setaria viridis* | 0.824 | 0.206 |
| *Sporobolus martitimus* | 1.422 | 0.079 |
| *Yushania levigata* | 1.771 | 0.040 |
